# Plant Diversity Reduces the Risk of Antibiotic Resistance Genes in Agroecosystems

**DOI:** 10.1002/advs.202410990

**Published:** 2025-01-28

**Authors:** Shu Li, Xing Zhou, Liangliang Liu, Zhe Su, Jun Zhao, Jinbo Zhang, Zucong Cai, Josep Peñuelas, Xinqi Huang

**Affiliations:** ^1^ School of Geography Nanjing Normal University Nanjing 210023 China; ^2^ CREAF Cerdanyola del Vallès Catalonia 08193 Spain; ^3^ Jiangsu Engineering Research Center for Soil Utilization & Sustainable Agriculture Nanjing 210023 China; ^4^ Jiangsu Center for Collaborative Innovation in Geographical Information Resource Development and Application Nanjing 210023 China

**Keywords:** antibiotic resistance genes, plant diversity, root exudates, soil microbial community

## Abstract

Despite advances in dispersal mechanisms and risk assessment of antibiotic resistance genes (ARGs), how plants influence ARG contamination in agricultural soils remains underexplored. Here, the impacts of plant species and diversity on ARGs and mobile genetic elements (MGEs) in three agricultural soils are comprehensively investigated in a pot experiment. The results indicate that increased plant diversity reduces ARGs and MGEs abundance by 19.2%–51.2%, whereas plant species exhibit inconsistent and soil‐dependent effects. Potential bacterial hosts harboring abundant ARGs have greater relative abundance than nonhosts, and both their richness and cumulative relative abundance are reduced by plant diversity. Notably, hosts inhibited by plant diversity present a greater relative abundance than the other hosts. The enriched compounds in root exudates due to plant diversity play a more important role in the metabolic network and contribute to rebalancing of the abundance of potential hosts and nonhosts. An independent test using pure organics reveals that higher resource diversity, resulting from increased plant diversity, reduces the relative abundance and mobility of abundant and high‐risk ARGs. This study highlights the resource‐mediated mitigation of the risks posed by ARG contamination and indicates that ensuring plant and resource diversity is a promising strategy for controlling ARGs in agroecosystems.

## Graphical abstract

There is a knowledge gap regarding how to effectively control antibiotic resistance genes (ARGs) and mitigate their health risks in agroecosystems. This study highlights the resource‐mediated mitigation of the risks posed by preexisting ARG contamination, indicating that ensuring the diversity of crops and resources is a promising strategy for controlling ARGs in agroecosystems.

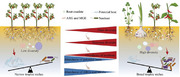

## 1 Introduction

Antibiotics, one of the most important medical discoveries of the 20^th^ century, have saved numerous lives and contributed significantly to controlling anthroponoses.^[^ ^1^ ^]^ In addition to their use in medicine, antibiotics have wide applications in the livestock production for disease prevention.^[^ ^2^ ^]^ However, antibiotics, as a competitive microbial strategy, were originally discovered in soil due to intense microbial competition for limited nutrients and ecological niches.^[^ ^3^ ^]^ Moreover, the selective pressure of antibiotics leads to the emergence of antibiotic resistance as a robust defense mechanism.^[^ ^4^ ^]^ The widespread distribution of antibiotic‐resistant bacteria (ARB) and antibiotic resistance genes (ARGs) in soil environments has been demonstrated.^[^ ^2^ ^]^ The extensive use of antibiotics in society has further resulted in the increased prevalence of ARB and ARGs in environments associated with human activity.^[^ ^5^, ^6^ ^]^ Horizontal and vertical transfer of ARGs facilitates global dissemination, with multi‐ARG‐containing superbugs posing substantial threats to human and animal health.^[^ ^7^, ^8^ ^]^ One example is the deadly outbreak in Germany that was attributed to ARB‐contaminated food.^[^ ^9^ ^]^ It has been reported that more than 10% of bloodstream *Staphylococcus aureus* infections are caused by methicillin‐resistant strains in 15 European countries, with approximately 50% resistance rates.^[^ ^10^ ^]^ Antibiotic resistance is a major 21^st^‐century challenge to human health, and ARGs are considered emerging contaminants.^[^ ^5^, ^11^, ^12^ ^]^ The World Bank predicts that by 2050, antimicrobial resistance could cause more than 10 million deaths annually and economic losses exceeding US$100 trillion if no timely action is taken.^[^ ^13^ ^]^

In addition to clinical settings, wastewater treatment plants and intensive animal husbandry are primary ARG reservoirs.^[^ ^2^, ^14^, ^15^ ^]^ Notably, ARGs in farmland soils and plants have gained attention because of the potential transfer of human‐associated and natural microbe‐borne ARGs via food consumption.^[^ ^2^ ^]^ However, the significance and risks of ARG transfer through the food chain might be underestimated.^[^ ^16^ ^]^ An analysis of 1088 soil metagenomes revealed greater ARG abundance in agroecosystems than in nonagricultural habitats.^[^ ^17^ ^]^ The application of sludge and manure to improve soil fertility and crop yield enriches ARGs in farmland soils,^[^ ^5^ ^]^ establishing them as major reservoirs and hotspots for ARG spread.^[^ ^18^, ^19^, ^20^ ^]^ Herbicides and heavy metals also increase ARG enrichment in agroecosystems.^[^ ^21^, ^22^, ^23^ ^]^ Current reports have demonstrated that types and contents of antibiotics and MGEs in soil aggravate the enrichment of ARGs in soil.^[^ ^24^, ^25^ ^]^ In addition, soil physicochemical properties including nutrient content and pH are proven to be important in determining ARG abundance.^[^ ^26^ ^]^ Despite the understanding of ARG dispersal and risk assessment, there is still a knowledge gap regarding how to effectively control the spread of ARGs and mitigate human health risks.^[^ ^19^ ^]^ Exploring new antibiotics to address antibiotic resistance risks does not fundamentally resolve ARG and ARB enrichment.^[^ ^27^, ^28^ ^]^ As soil microbes in a narrow ecological niche fiercely compete for limited nutrients and space,^[^ ^29^ ^]^ ARB carrying multiple ARGs are likely to have competitive advantages and become dominant due to their antimicrobial resistance capacity.^[^ ^30^, ^31^ ^]^ Moreover, microbial competitive advantages are also influenced by available resources and trophic niche breadth.^[^ ^32^ ^]^ Thus, manipulating ARB competitive advantages through the regulation of nutrient access offers a potential strategy for suppressing ARB.^[^ ^32^ ^]^

Plants substantially impact soil microbial communities through root exudates, releasing approximately 5%–40% of photosynthetic products into the rhizosphere as a food source for microbes.^[^ ^33^, ^34^, ^35^, ^36^ ^]^ Different plant species and cultivars present unique exudate profiles, influencing rhizosphere microbial community composition and function.^[^ ^37^, ^38^, ^39^, ^40^ ^]^ Owing to their specific nutrient preferences, soil microbes, including ARB, likely have various competitive advantages under the influence of distinct plant species.^[^ ^41^ ^]^ The diversity of the soil resistome is significantly affected by plant species.^[^ ^42^ ^]^ On the other hand, long‐term monocropping decreases chemical resource diversity and narrows trophic niches, leading to a decrease in soil microbial diversity and specific microbes, including ARB, which results in competitive advantage.^[^ ^43^, ^44^ ^]^ Numerous reports indicate that aboveground biodiversity supports chemical resource diversity, which is crucial for belowground biodiversity.^[^ ^45^, ^46^ ^]^ Plant and chemical resource diversity can partition trophic niches, disrupt the competitive advantages typically held by dominant species, and increase microbial evenness.^[^ ^47^ ^]^ For example, Wang et al. reported that intercropping significantly increased microbial diversity and inhibited the growth of *Fusarium oxysporum*, which is dominant under monocropping.^[^ ^48^ ^]^ Furthermore, a global study revealed that 7137 human‐animal pathogenic amplicon sequence variants were detected in the rhizosphere soil microbiomes, and that their abundance was negatively associated with microbial diversity.^[^ ^49^ ^]^ Thus, plant identity and diversity likely possess strong potential to regulate the competitive advantages and abundance of ARB by controlling nutrient access and trophic niche breadth.

Overall, ARG contamination in agricultural soils, particularly in manure‐amended vegetable cropping soils, poses risks to human health.^[^ ^19^ ^]^ Knowledge of strategies to reduce the abundance of ARB and ARGs in agroecosystems is thus essential. The production of antibiotics is a competitive strategy for the acquisition of resources; thus, ARB capable of resisting antibiotics have greater capacity during resource competition.^[^ ^50^ ^]^ Hence, we hypothesize that ARB, particularly multi‐drug‐resistant bacteria, have competitive advantages over nonhost bacteria in a narrow trophic niche. The use of different crop species may alter the competitive advantages of ARB, as previous reports indicate that aboveground biodiversity supports chemical resource diversity and distinct trophic niches.^[^ ^51^ ^]^ Crop diversity that induces resource diversity may reduce ARB competitive advantage through niche partitioning, consequently decreasing ARB and ARG abundance. Here, we tested our hypotheses by investigating the impacts of crop type and diversity on ARG abundance by planting various crop species individually or in combination in ARG‐contaminated agricultural soils (Figure S1, Supporting Information). Three soil types with distinct properties were employed to assess the consistency of plant effects on ARGs. To verify the effect of plant diversity that induced resource diversity on reducing ARG contamination, an independent experiment was further conducted by using pure chemicals from root exudates to create a resource diversity gradient.

## 2 Results

### 2.1 Effects of Plant Diversity and Species on the Abundance of ARGs

**Figure 1:**
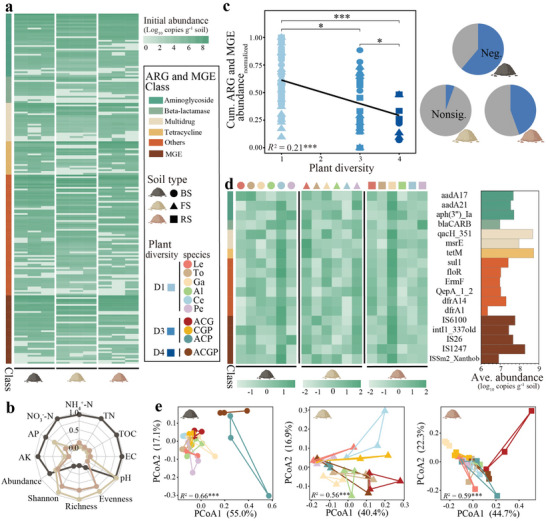
Abundance of antibiotic resistance genes (ARGs) and mobile genetic elements (MGEs) in soils cultivated with different plants. a) Initial abundance of the 200 most abundant ARGs and MGEs, determined via high‐throughput real‐time PCR, in black soil (BS), fluvo‐aquic soil (FS), and red soil (RS). b) Initial physicochemical and microbial properties of the BS, FS, and RS. The data were max‐min normalized within all soils. c) Response of the normalized cumulative abundance of the 18 abundant ARGs and MGEs to plant diversity in each soil. The data were max‐min normalized within each soil type, and the significance was detected by one‐way ANOVA and Duncan's test. The *R^2^* and *p*‐value were determined by the general linear model. The pie chart indicates the proportion of ARGs and MGEs that are negatively (blue) or not significantly (gray) correlated with plant diversity. d) Abundance of ARGs and MGEs in each soil after the cultivation of different plant species. The right bar indicates the average abundance of each ARG or MGE across all soils. e) Principal coordinate analysis, using the Bray‐Curtis distance, of the composition of ARGs and MGEs in each soil type under different planting patterns. The *R^2^* and *p*‐value were determined by PERMANOVA. *, and *** represent *p* < 0.01, and 0.001, respectively.

Plants of different species and diversities were planted in ARG‐contaminated black soil (BS), fluvo‐aquic soil (FS), and red soil (RS) for seven months (Figure S1, Supporting Information). We employed high‐throughput qPCR to comprehensively investigate the initial abundance of ARGs and mobile genetic elements (MGEs) in the tested soils. The three most abundant classes of ARGs were the aminoglycoside, tetracycline, and multidrug classes (**Figure** **1**a). The cumulative abundance of ARGs was nearly equivalent among the three soils, while the abundance of MGEs in the BS and RS was significantly (*p* < 0.05) greater than that in the FS (Figure S2, Supporting Information). The contents of total organic carbon (TOC), total nitrogen (TN), and inorganic nitrogen in FS were lower than those in both BS and RS (Figure 1b; and Table S1, Supporting Information). In contrast, compared with those in the other soils, the bacterial richness, evenness, and Shannon diversity in the FS soils were greater (Figure 1b; and Table S1, Supporting Information).

After a seven‐month planting period, we determined the abundance of 18 prevalent ARGs and MGEs across various planting patterns. A significant (*p* < 0.001) negative correlation was observed between the cumulative abundance of ARGs and MGEs and plant diversity in the three soils (Figure 1c). Additionally, high‐risk ARGs significantly decreased with increasing plant diversity (Figure S3, Supporting Information). Compared with those in the monoculture treatment, the cumulative abundance of ARGs and MGEs in the high plant diversity treatment decreased by 51.2%, 19.2%, and 19.3% in the BS, FS, and RS, respectively. Two‐way ANOVA of plant diversity and soil type revealed that plant diversity significantly (*p* < 0.001) affected ARG and MGE abundance (Table S2, Supporting Information). Specifically, 61.1%, 5.6%, and 44.4% of the ARGs and MGEs were significantly (*p* < 0.05) reduced in terms plant diversity in the BS, FS, and RS, respectively (Figures S4–S6, Supporting Information). With respect to the impact of plant species, the celery, lettuce, and garlic treatments yielded the highest cumulative abundance of ARGs and MGEs in the three soils, whereas the lowest values were observed after the alfalfa, tomato, and alfalfa treatments, respectively (Figure 1d; and Figure S7, Supporting Information). These results highlight the inconsistent and soil‐specific impacts of plant species on ARG and MGE abundance.

Furthermore, the influence of plant diversity and species on ARG and MGE composition was investigated, revealing significant effects of plant diversity (*p* < 0.001, *p* > 0.05, *p* < 0.01 according to permutational multivariate analysis of variance (PERMANOVA), respectively) and species (*p* < 0.05, *p* < 0.001, *p* < 0.001 according to PERMANOVA, respectively) in the three distinct soils (Figure 1e; Figure S8, Supporting Information). Moreover, the influence of plant diversity in the BS and RS exceeded that in the FS, whereas the impact of plant species in the BS was lower than that in the other soils (Figure S8, Supporting Information).

### 2.2 Effects of Plant Diversity and Species on the Bacterial Community

**Figure 2:**
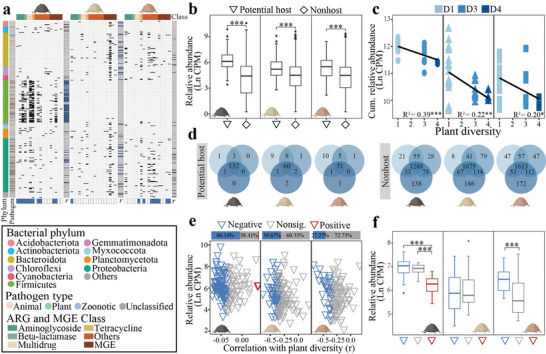
Potential hosts of the 18 abundant antibiotic resistance genes (ARGs) and mobile genetic elements (MGEs) and their relationships with plant diversity. a) Potential hosts in each soil identified by Spearman analysis with thresholds of *ρ* > 0.6 and *p* < 0.01. The pathogenicity of these potential hosts was identified via multiple bacterial pathogen detection. The colors in the right column and the bottom row labeled with “*r”*, corresponding to each potential host or ARG and MGE, indicate a positive (red), negative (blue), or nonsignificant (gray) relationship with plant diversity. b) Relative abundance of the identified potential hosts and nonhosts in each soil type. CPM denotes counts per million. c) Responses of the cumulative relative abundance of the potential hosts to plant diversity. The *R^2^* and *p*‐value were determined by the general linear model. d) Venn diagram showing the numbers of soil bacterial OTUs affiliated with the potential hosts and nonhosts under different plant diversities. The OTUs present in more than 2/3 of the samples of each diversity gradient were retained for analysis. e) Relationships between the relative abundance of potential hosts and plant diversity in each soil type determined via Pearson correlation analysis. f) The average relative abundance of the potential hosts in the negative, positive, and nonsignificant groups. The significance was detected by one‐way ANOVA and Duncan's test in [b] and [f]. *, **, and *** represent *p* < 0.05, 0.01, and 0.001, respectively.

To elucidate the regulation of ARGs and MGEs by plant diversity, we first identified potential hosts through correlations between OTU abundance and ARG and MGE abundance. Given the substantial variations among the three soils, potential host identification was conducted separately for each soil. In the BS, 76.09% of the potential hosts were affiliated with the phyla Firmicutes and Proteobacteria, while the phyla Bacteroidota and Proteobacteria were dominant for potential hosts in the FS and RS (**Figure** **2**a). Most of these potential hosts are pathogenic to animals and plants. Moreover, the relative abundance of potential hosts was significantly (*p* < 0.001) greater than that of nonhosts in all soil types (Figure 2b), indicating a clear competitive advantage for potential hosts. A significant (*p* < 0.05) decrease in both the richness and cumulative abundance of potential hosts was observed with increased plant diversity (Figure 2c; Figure S9, Supporting Information). Additionally, the number of unique OTUs in the nonhost community increased with increasing plant diversity, whereas the number of unique OTUs in the potential host community decreased (Figure 2d).

Analysis of potential hosts in each soil type revealed that the relative abundance of 60.14%, 39.67%, and 27.27% of potential hosts were negatively correlated with plant diversity (Figure 2e). Furthermore, the average relative abundance of the negative group was greater than that of both the nonsignificant and positive groups in the BS and RS (Figure 2f). Additionally, a positive correlation between ARG and MGE numbers in potential hosts and their relative abundance was observed (Figure S10, Supporting Information). These results indicated that plant diversity tended to suppress potential hosts with higher abundance and more ARGs and MGEs.

We further compared the impacts of plant diversity on the total, potential host, and nonhost bacterial communities. Plant diversity significantly (*p* < 0.05) influenced all the structures of all community types (Figure S11, Supporting Information). Furthermore, the impact of plant diversity on the potential host community was greater in the BS (*R* ^2^ = 0.29) than in the other soil types (*R* ^2^ = 0.18 and 0.16). Biomarkers of plant diversity within the potential host and nonhost communities were identified, with most potential host community biomarkers inhibited by plant diversity and most nonhost community biomarkers promoted by plant diversity (Figure S12, Supporting Information). Moreover, microbial connections strengthened with increasing plant diversity, as evidenced by increased total edges and the proportion of negative edges with increasing plant diversity (Figure S13, Supporting Information). Additionally, plant species significantly (*p* < 0.001) impacted both the structure (Figure S14, Supporting Information) and composition (Figure S15, Supporting Information) of the total, potential host, and nonhost bacterial communities.

### 2.3 Effects of Plant Diversity and Species on Root Exudates

**Figure 3:**
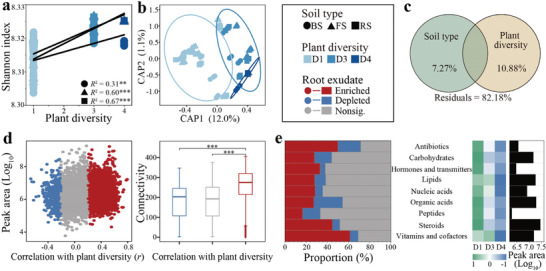
Changes in root exudates with increasing plant diversity. a) Response of the Shannon diversity index of root exudates to plant diversity in each soil. The *R^2^* and *p*‐value were determined by the general linear model. b) Constrained analysis of principal coordinates (CAP) of root exudate profiles under different plant diversities. c) Contributions of soil type and plant diversity to the variation in root exudate profiles. d) Peak areas and connectivity indices of the nonsignificantly affected, enriched and depleted root exudate components according to plant diversity. The components significantly (*p* < 0.05) enriched and depleted by plant diversity are detected by Pearson correlation analysis and shown in red and blue, respectively. The significance in the connectivity was detected by one‐way ANOVA and Duncan's test. e) Relationships between well‐classified compounds and plant diversity. The left panel shows the proportions of the enriched, depleted and nonsignificantly affected compounds by plant diversity within each class. The right heatmap indicates the peak area of each class (cumulative peak area of all compounds in this class) in different plant diversity gradients, and the bar denotes the average peak area of this class across all soils. The color in the heatmap indicates the z score‐normalized peak area. **, and *** represent *p* < 0.01, and 0.001, respectively.

As anticipated, the Shannon diversity of the root exudates was significantly (*p* < 0.01) positively correlated with plant diversity across all the soils (**Figure** **3**a). The planting pattern, which represents the difference in plant diversity and identity, and the soil type significantly (*p* < 0.001 according to PERMANOVA) affected the root exudate profiles (Figure S16a, Supporting Information), explaining 68.33% and 9.44% of the observed variation, respectively (Figure S16b, Supporting Information). Plant diversity significantly (*p* < 0.001) influenced the composition of the root exudates (Figure 3b), accounting for 10.88% of the observed variation (Figure 3c). Among the 4227 compounds detected, 43.48% and 9.87% of the compounds in the root exudates were significantly enriched and depleted, respectively, by increasing plant diversity (Figure 3d).

The connectivity of enriched compounds, determined via network analysis of root exudate compounds (Figure S17, Supporting Information), was significantly (*p* < 0.001) greater than that of depleted and not significantly affected compounds, indicating the important role of these enriched compounds in plant metabolic pathways (Figure 3d). Moreover, we identified feature compounds associated with plant diversity and observed that most of these compounds were enriched by plant diversity (Figures S18 and S19, Supporting Information). Among the 190 compounds that were well classified into nine categories, the proportion of enriched compounds exceeded that of depleted compounds, except for the organic acid and peptide categories (Figure 3e). A decreasing trend in cumulative peak areas associated with plant diversity was noted across all categories (Figure 3e; Figure S20, Supporting Information). Furthermore, significant effects of plant species on the Shannon diversity, structure, and composition of root exudates were also observed (Figures S21 and S22, Supporting Information).

### 2.4 Linkages Among Plant Diversity, Root Exudates, Potential Hosts, ARGs, and MGEs

**Figure 4:**
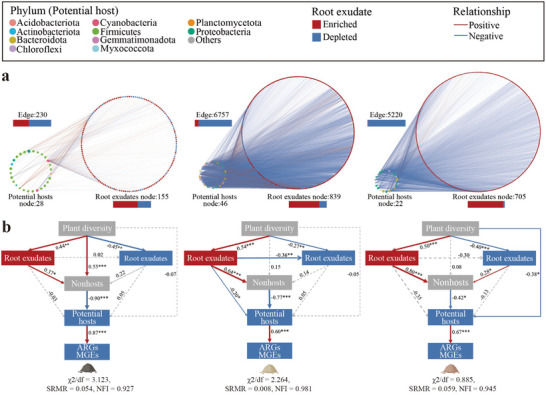
Linkages among plant diversity, root exudates, potential hosts, antibiotic resistance genes (ARGs) and mobile genetic elements (MGEs). a) Network analysis showing the relationships between the abundance of the potential host and those significantly (*p* < 0.01) altered root exudate components according to plant diversity. Significant (*p* < 0.01) and close (|*ρ*| > 0.7) edges based on Spearman rank correlation analysis were retained. The bars denote the proportions of the enriched and depleted components, as well as the proportions of negative and positive links between the root exudates and the potential hosts. b) Partial least square‐structural equation modeling showing the pathway by which plant diversity decreases the abundance of ARGs and MGEs. The cumulative values of the significantly (*p* < 0.01) enriched and depleted root exudates, the potential hosts and nonhosts, and the significantly (*p* < 0.05) reduced ARGs and MGEs according to plant diversity were used for the analysis. The background color of each variable denotes its relationship with plant diversity. The significance was detected by bootstrapping in smartPLS.

To elucidate how plant diversity reduces the abundance of potential hosts and ARGs and MGEs through the regulation of root exudates, we analyzed the relationships between potential hosts and compounds significantly affected by plant diversity. The results revealed that 58.26%, 89.42%, and 98.52% of the relationships observed were negative in the BS, FS, and RS, respectively (**Figure** **4**a). The application of partial least squares structural equation modeling revealed causal pathways through which plant diversity decreased the abundance of ARGs and MGEs. Specifically, the enriched compounds in the root exudates stimulated nonhosts, inhibiting potential hosts and leading to a decrease in the abundance of ARGs and MGEs (Figure 4b). These findings suggest that plant diversity plays a regulatory role in the competitive advantage between nonhosts and potential hosts by enriching specific compounds and increasing the chemical diversity of root exudates.

### 2.5 Effects of Resource Diversity on ARG Abundance in the Independent Validation Test

**Figure 5:**
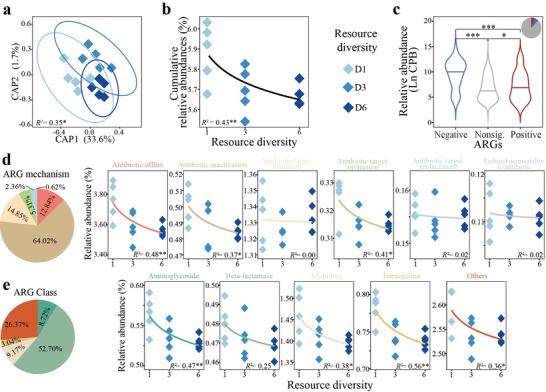
Responses of antibiotic resistance genes (ARGs) to resource diversity in the independent validation test. a) Constrained analysis of principal coordinates (CAP) of soil ARG composition following resource diversity using the Bray‐Curtis distance. The *R^2^* and *p*‐value were determined by PERMANOVA. b) Response of the cumulative relative abundance of ARGs to resource diversity. c) Relative abundance of the ARG groups for which abundance was negatively, positively, or nonsignificantly affected by resource diversity. CPB denotes count per billion. The significance was detected by one‐way ANOVA and Duncan's test. The pie chart at the top‐right corner shows the proportions of the ARG number in the three groups. d) and e) Proportions of ARGs affiliated with different resistance mechanisms (d) and classes (e) and their relationships with resource diversity. The *R^2^* and *p*‐value were determined by the logarithmic fitting model in [b], [d], and [e]. *, **, and *** indicate *p* < 0.05, 0.01, and 0.001, respectively.

To further support the hypothesis that increased resource diversity decreases ARG abundance, a validation test was conducted. Pure organic substances with varying levels of diversity were introduced into soil contaminated with ARGs. Metagenomic sequencing revealed a total of 2203 ARGs, and the ARG composition was significantly (*p* < 0.05) influenced by resource diversity (**Figure** **5**a). In line with previous findings, the cumulative relative abundance of ARGs significantly (*p* < 0.01) decreased with increasing resource diversity (Figure 5b). Compared with that in the single‐source treatment, the resource diversity decreased by 3.55% in the resource diversity treatment containing six sources. Specifically, 7.31% of the ARGs presented a negative correlation with resource diversity, whereas 4.36% presented a positive correlation. The effects of resource diversity on ARG composition and abundance were greater than those of singular resources (Figure S23, Supporting Information). In addition, resource diversity significantly (*p* < 0.05 according to PERMANOVA) affected the microbial communities (Figure S24, Supporting Information).

Furthermore, the relative abundance of negative ARGs was significantly (*p* < 0.001) greater than that of positive and nonsignificant ARGs (Figure 5c). ARGs were further classified on the basis of resistance mechanisms and drug classes, revealing that ARGs associated with antibiotic efflux, inactivation, and target protection decreased significantly (*p* < 0.05) with increasing resource diversity (Figure 5d). All classes of ARGs were reduced in terms of resource diversity, although the beta‐lactamase class did not significantly differ (Figure 5e). Compared with those in the single‐source treatment, the relative abundance of several high‐risk ARG classes, i.e., aminoglycoside, multidrug, and tetracycline, decreased by 6.30%, 4.32%, and 5.49%, respectively, in the resource diversity treatment containing six sources.

**Figure 6:**
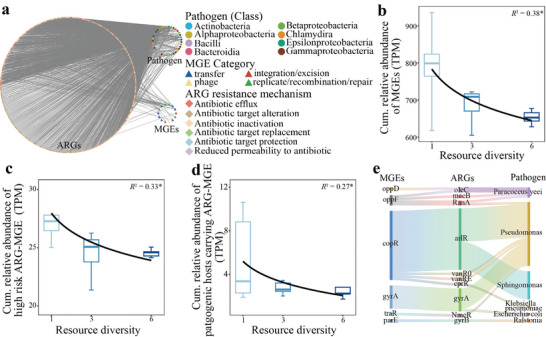
Impact of resource diversity on the mobility of antibiotic resistance genes (ARGs) and pathogenic hosts of ARGs in the independent validation test. a) Cooccurrence network for ARGs, mobile genetic elements (MGEs), and pathogenic bacteria. b) Response of the cumulative relative abundance of MGE‐carrying contigs to resource diversity. c) Response of the cumulative relative abundance of high‐risk ARG‐MGE carrying contigs to resource diversity. High‐risk ARGs were identified according to the criteria of human accessibility, mobility, pathogenicity and clinical availability. d) Response of the cumulative relative abundance of the pathogenic hosts carrying both ARGs and MGEs to resource diversity. e) Proportions of pathogenic bacteria carrying both ARGs and MGEs. The *R^2^* and *p*‐value were determined by the logarithmic fitting model in [b], [c], and [d]. The symbol * indicates *p* < 0.05.

The mobility of ARGs and the pathogenicity of their hosts were further investigated (**Figure** **6**a). At the class level, *Alphaproteobacteria* (79.90%) and *Gammaproteobacteria* (18.06%) were the two dominant bacterial classes among the pathogenic hosts. Most MGEs that cooccurred with ARGs belonged to the transfer (61.19%) and replication/recombination/repair (36.67%) categories. Although multiple MGEs were detected, only 469 ARG‐carrying contigs carried MGEs. The cumulative relative abundance of MGEs and high‐risk ARG‐MGE carrying contigs significantly (*p* < 0.05) decreased as resource diversity increased (Figure 6b,c). Furthermore, we investigated pathogenic bacteria carrying both ARGs and MGEs and found that the relative abundance of those bacteria posing a high‐risk to humans was also suppressed by increasing resource diversity (Figure 6d,e).

## 3 Discussion

Mounting evidence underscores the pivotal role of plants in shaping soil microbial diversity, composition, and multifunctionality.^[^ ^52^, ^53^ ^]^ However, the understanding of the influence of plants on ARGs in agroecosystems remains limited, despite the substantial risks they pose to human health.^[^ ^17^ ^]^ Recent research has shown that plant species exert a significant effect on nematode resistome abundance.^[^ ^42^ ^]^ In the context of agricultural soils, a systematic analysis revealed greater ARG abundance in soils planted with pak choi than in soils planted with other vegetable types.^[^ ^19^ ^]^ In alignment with these findings, our study also revealed a significant impact of vegetable type on soil ARG abundance. Nonetheless, the influence of plant species on ARG abundance was not consistent across different soil types. The presence of soil type‐dependent effects underscores the complexity of achieving consistent control of ARG contamination via specific plant species. In contrast, our results revealed a consistent reduction in ARG abundance with increasing plant diversity across most soils. The application of livestock manure in agroecosystems is widely recognized as the primary reason for the enrichment of ARGs in farmland soils,^[^ ^54^ ^]^ and our results suggest that the reduction in plant diversity in agroecosystems may be another reason accounting for the high level of soil ARGs. To our knowledge, this study is the first to propose that increasing aboveground biodiversity is a promising avenue for mitigating soil ARG contamination.

Theoretical considerations suggest that soil‐borne ARB, especially those harboring multiple ARGs, are advantageous for intense competition with microbes that have similar nutrient and niche preferences. This advantage stems from the production of antibiotics, a pivotal strategy for outcompeting microbial rivals.^[^ ^1^ ^]^ Experimental evidence confirms that a greater number of ARGs leads to increased gene expression and improved cell survival.^[^ ^55^ ^]^ Similarly, our findings revealed a significant increase in the relative abundance of ARG‐hosts carrying abundant ARGs across all soil types (Figure 2b). Moreover, a positive and significant correlation existed between the number of ARGs possessed by a host and its relative abundance (Figure S10, Supporting Information). These results provide additional support for the competitive advantage of soil bacteria harboring abundant and diverse ARGs. Thus, the quantification of ARGs can serve as a reliable indicator of species competition, alongside genes linked to resource acquisition and antibiotic production.^[^ ^30^ ^]^ Moreover, owing to a lack of specificity in the relationship between ARGs and their hosts, which increases the possibility of horizontal transfer of ARGs to phylogenetically unrelated bacteria,^[^ ^56^ ^]^ different soils harbor distinct microbial communities and ARG hosts.^[^ ^54^, ^57^, ^58^ ^]^ This finding aligns with our own, as we discovered wide variability in potential hosts among the three soil types. This variability might also account for the inconsistent effects of plant species on ARG abundance across these soil types, given the influence of plant species on specific microbes.^[^ ^59^ ^]^

With respect to the influence of plant diversity, both the richness and cumulative relative abundance of potential hosts decreased significantly with increasing plant diversity, whereas those of nonhosts increased significantly. These observations suggest that plant diversity functions as a homogenizing force in soil microbial communities by favoring nonhosts with lower relative abundance and suppressing potential hosts with higher relative abundance. Furthermore, we found that the decrease in the relative abundance of potential hosts with increasing plant diversity was greater than that of unaffected and increased hosts, underscoring the homogenizing impact of plant diversity. Aboveground plants primarily regulate soil microbes through the production of root exudates, which are rich in organic resources.^[^ ^60^, ^61^ ^]^ Recent studies have emphasized the importance of resource availability in governing microbial competition and community assembly.^[^ ^62^ ^]^ Plant diversity increases resource diversity, which prompts trophic niche partitioning,^[^ ^63^ ^]^ weakens the competitive advantage of potential hosts, mitigates the imbalance between abundant hosts and relatively rare nonhosts, and complicates the microbial network. This pattern aligns with findings from our previous study, where increased resource diversity led to greater microbial evenness and connectivity.^[^ ^47^ ^]^

Previous studies have shown that soil organic matter and nutrients are important factors influencing ARGs.^[^ ^64^ ^]^ Gao et al. reported a negative relationship between soil C:N and ARGs within wheat and cucumber cropping systems.^[^ ^26^ ^]^ Fu et al. reported that the content of soil organic matter mainly affected the relative abundance of ARGs in grassland and cropland.^[^ ^65^ ^]^ These findings align with our results, indicating that the three different soils contained distinct ARGs and MGEs. An increase in plant diversity led to a greater decrease in the cumulative abundance of ARGs in the BS than in the other soils (Figure 1c). Additionally, the potential hosts in the BS had greater richness and average relative abundance than those in the FS and RS (Figure 2b,d). These findings implied that the potential host in the BS had a superior competitive advantage in comparison with other soils, which was correlated with the lowest microbial evenness and highest nutrient content observed in the BS. Consequently, these outcomes imply that the homogenizing influence of plant diversity on soil microbes is greater in fertile soils, which exhibit relatively low microbial evenness. This variability might contribute to the inconsistency observed in the relationship between plant diversity and belowground diversity across different soil types.^[^ ^66^ ^]^

To investigate how plant diversity affects root exudates and subsequently impacts the abundance of potential hosts and ARGs, we analyzed root exudate profiles under various planting patterns. As expected, increased plant diversity yielded greater organic resource diversity, particularly emphasizing evenness. Different compounds exhibited varied responses to plant diversity, demonstrating both enrichment and depletion, which aligns with prior research.^[^ ^67^ ^]^ While specific compounds within root exudates directly inhibit the growth of particular soil microbes,^[^ ^68^ ^]^ in many instances, certain root exudates tend to stimulate the growth of distinct microbial groups.^[^ ^69^ ^]^ Our findings illustrate that increased plant diversity indirectly restrains potential hosts primarily by stimulating nonhosts. Moreover, enriched compounds with greater connectivity play pivotal roles in metabolic pathways and contribute to balancing potential hosts and nonhosts in this study.^[^ ^70^ ^]^

An independent study further validated our observations by introducing pure chemicals with a diversity gradient into the soil, substantiating that resource diversity substantially reduces soil ARG abundance, especially highly prevalent ARG abundance. This finding provides additional evidence of how plant diversity modulates competition between potential ARG hosts and other microbes. Notably, not all ARGs pose substantial threats to public health.^[^ ^71^ ^]^ A recent study assessing risk using metagenomic data from 4572 samples highlighted the health risks posed by multidrug resistance.^[^ ^6^ ^]^ Encouragingly, our findings revealed that increased resource diversity significantly decreased the prevalence of multidrug resistance genes. Moreover, antibiotic efflux, a pivotal mechanism in multidrug resistance,^[^ ^27^ ^]^ displayed heightened sensitivity to resource diversity within all ARG categories. In addition, the health risks associated with ARGs were thoroughly evaluated by considering both the mobility of ARGs and the pathogenicity of their hosts. We found that increased resource diversity effectively reduced the mobility of high‐risk ARGs and their pathogenic hosts, which is crucial for mitigating the health risks of ARGs in soil.^[^ ^72^ ^]^ In addition, it should be acknowledged that the pure carbohydrates serve only as a proof of concept regarding the effects of resource divesity on soil ARGs, and it cannot represent in vivo rhizodeposits which are more complex.

## 4 Conclusion

**Figure 7:**
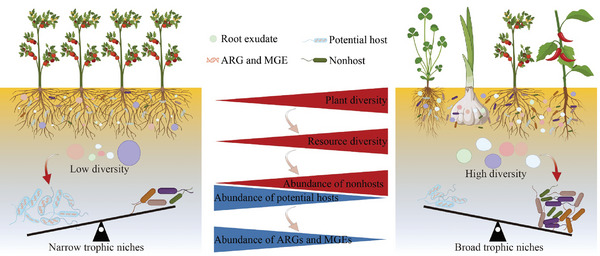
Conceptual model illustrating that plant diversity regulates the competitive advantages between antibiotic resistance gene (ARG) hosts and nonhosts.

This study provides novel insights by revealing that the increase in resource diversity, which results from increased plant diversity, substantially decreases the abundance of soil ARGs in agroecosystems. The reduction in plant diversity in agroecosystems compared with that in natural ecosystems is one of the reasons for the greater level of ARGs in agricultural soils. Microbes endowed with antibiotic resistance attributes gain a competitive edge, leading to their increased prevalence in soils (**Figure** **7**). The increase in plant diversity regulates competition dynamics between potential hosts of ARGs and nonhosts via trophic niche partitioning. The root exudate‐enriched compounds, stemming from increased plant diversity, have greater connectivity and stimulate the growth of underprivileged nonhost bacteria, effectively suppressing potential hosts. ARB and ARGs displaying elevated abundance were notably susceptible to the homogenizing influences of both plant and resource diversity. The high‐risk ARG categories, including multidrug resistance and antibiotic efflux, experienced significant reductions due to increased resource diversity. Notably, resource diversity mitigates the potential risks of ARGs in soil by reducing the presence of ARG‐MGE carrying contigs and their pathogenic hosts. Besides, this study was primarily founded on pot experiments using three types of soils, which may possess certain limitations in reflecting the complex circumstances in the field. Although the validity of these inferences has been partially confirmed by the independent experiments, future investigations are necessary to further explore the effects of available resources on the competitive advantage of ARB and the migratory patterns of ARGs, particularly under field conditions. This could contribute to a better understanding of the dynamics of ARGs in heterogeneous environments. Overall, our findings offer a pathway for suppressing the competitive advantage of ARG hosts through trophic niche partitioning. We propose that augmenting plant diversity holds promise as a robust strategy for mitigating the risks posed by prevalent ARG contamination, particularly in agroecosystems where concerns regarding public health are important owing to their close association with human activity.

## 5 Experimental Section

### Experimental Design

Three types of soils were collected contaminated with ARGs that had a history of long‐term manure application. These soils included BS, FS, and RS. BS was collected from a long‐term corn‐tomato rotation field in Changchun city (44° 25′ N, 125° 9′ E), Jilin Province, where pig manure and sludge were applied regularly. FS was collected from farmland in Nantong city (32° 39′ N, 120° 42′ E), Jiangsu Province, where cabbage, potato, and maize were rotated and fresh chicken manure was used. RS was collected from a fallow land in Jian city (26° 57′ N, 114° 14′ E), Jiangxi Province, where pig manure has been piled for a long period. These soils were sieved to remove stones and impurities and subsequently homogenized.

A monoculture study was conducted using six crops: lettuce, tomato, garlic, alfalfa, celery, and pepper. The purpose of this study was to investigate how plant species influence the regulation of soil microbial communities and ARGs. Additionally, to establish a plant diversity gradient, combinations of plant species were selected. These combinations included three randomly chosen species from among garlic, alfalfa, celery, and pepper, as well as all four species together. This resulted in a total of 10 planting patterns, comprising six monoculture patterns, three patterns with combinations of three species, and one pattern with a combination of all four species. Each planting pattern was replicated three times for each soil type, resulting in a total of 90 pots (3 soil types × 10 planting patterns × 3 replicates). Each soil type, in amounts of six kilograms, was mixed with 2 L of sterile vermiculite and perlite. Each mixture was then packed into pots measuring 45 cm × 34 cm × 10 cm. One‐month‐old seedlings were subsequently transplanted into these pots. Over the course of two planting seasons, for a total of seven months, the soil in each pot placed in a greenhouse was irrigated monthly with 500 mL of a 1/4 Hoagland nutrient mixture, with temperatures fluctuating between 22 °C and 35 °C. Consistent plant biomass levels were maintained across all pots in each soil type by adjusting the planting density of each species, and the biomass of individual plant species in pots with multiple species was approximately equal.

After each season, the root exudates from all the plant samples in each pot were collected via an established method.^[^ ^73^ ^]^ Briefly, the soil adhering to the plant roots was carefully washed with distilled water. The plants were cultivated in 1/2 Hoagland's solution for 24 h at 25 °C with 14 h of light/10 h of darkness. The solution was subsequently freeze‐dried and stored at −80 °C. The root exudates from the same pot collected across both seasons were combined. Hoagland's solution without plants was used as a negative control. Following a seven‐month cultivation period, soil samples were collected from each pot by blending five subsamples at different locations. These collected samples were then sieved, homogenized, and stored at −80 °C for future use.

To validate the impact of resource diversity on reducing ARG abundance, an independent test was conducted according to the design described by Zhou, et. al.^[^ ^47^ ^]^ The physicochemical properties of the test soil were as follows: TOC 18.7 g kg⁻¹, TN 2.17 g kg⁻¹, available phosphorus (AP) 35.52 mg kg⁻¹, available potassium (AK) 335.67 mg kg^−1^, and pH 7.52. Briefly, the tomato planting soil was irrigated periodically (every five days) with organic carbon mixtures of the same concentration and varying amounts of carbohydrates, with a final concentration of 0.5 mg g^−1^ soil each time. The carbon mixtures were created by randomly selecting one, three, and six carbohydrates from a pool of 12 different carbohydrates (Table S5, Supporting Information). The concentration of each carbohydrate was equal within each carbon mixture. This random selection process was repeated five times for each resource gradient. In total, a set of 15 pots (45 cm × 34 cm × 10 cm) was utilized. Each pot was filled with eight kilograms of soil, and nine tomato plants were planted. After 90 days of cultivation, metagenomic sequencing was performed on the soil samples collected by combining nine subsamples from each pot.

### Determination of Soil Physicochemical Properties

Soil electrical conductivity (EC) and pH were measured at soil‐to‐water ratios of 1:5 and 1:2.5 (w/v), respectively, using S220 and S230 meters (Mettler‐Toledo International Inc., Shanghai, China). Soil NH_4_^+^‐N and NO_3_^‐^‐N were extracted with a 2 m KCl solution at a soil‐to‐solution ratio of 1:5 (w/v) and quantified using a continuous flow analyzer (San++, Skalar Analytical, Breda, The Netherlands). The soil TOC, and TN contents were determined following established methods.^[^ ^74^, ^75^ ^]^ Soil AP, and AK were extracted using 0.5 m NaHCO_3_ and 1 m CH_3_COONH_4_ solutions and quantified using the molybdenum‐antimony colorimetric method and flame photometry, respectively.

### Quantification of ARGs and MGEs

Soil DNA was extracted using the FastDNA Spin Kit (MP Biomedicals, Santa Ana, CA, USA) following the manufacturer's protocol. The concentration and purity of the extracted soil DNA were measured using a DS‐11 spectrophotometer (Denovix Inc., Wilmington, DE, USA). High‐throughput quantitative polymerase chain reaction (HT‐qPCR) was conducted using SmartChip Real‐time PCR (Wafergen Inc. Fremont, CA, USA) to determine the initial abundance of ARGs and MGEs in the three soils. The sequences of 367 primer sets targeting 309 ARGs, 57 MGEs, and 1 bacterial 16S rRNA gene are provided in Table S3 (Supporting Information). Sterile water and a mixed solution of standard plasmids containing all target genes as templates served as the negative and positive controls, respectively. The amplification procedure followed a previous report.^[^ ^76^ ^]^ Each amplification was performed in triplicate, and the amplification efficiency for each gene was maintained within the range of 1.8–2.2. The relative copy number of ARGs and MGEs was calculated according to the method described by Looft, et al. using the following formula: relative gene copy number = 10^[(31–CT) / (10/3)].^[^ ^76^, ^77^ ^]^ CT represents the cycle threshold of quantitative PCR results, and 31 indicates the detection limit threshold. Subsequently, the relative copy number was converted into absolute copy number by normalizing it to the absolute copy number of the 16S rRNA gene. The quantification data for genes detected in all three replicates were retained for further analysis. On the basis of the initial abundance of ARGs and MGEs in the three soils, 5 abundant MGEs and 13 abundant ARGs, including aminoglycosides, beta‐lactams, tetracyclines, and others with high amplification specificity, were selected for detection in the soil samples after plant cultivation. The abundance of these 18 abundant ARGs and MGEs covaries (*R* ^2^ = 0.92) and accounts for 39.4% of the total abundance of ARGs and MGEs (Table S4, Supporting Information). According to the ARG risk levels classified by a framework proposed by Zhang, et al.,^[^ ^6^ ^]^ eight (*aadA17, msrE, tetM, sul1, floR, ErmF, dfrA14*, and *dfrA1*) of the 18 ARGs and MGEs were classified as high‐risk ARGs. ARG risk levels is based on considerations of human accessibility, mobility, pathogenicity, and clinical availability. Human accessibility refers to the potential transmission of ARGs from the environment to bacteria in humans. Mobility and human pathogenicity encompass the potential transfer of ARGs from environmental bacteria to human pathogens, considering that only ARGs present in pathogenic hosts that could pose an elevated risk to human health. The clinical availability of ARGs correlates with the current clinical use of antibiotics. qPCR detection was conducted via a QuanStudio 3 Real‐time PCR system (Applied Biosystems, Foster City, CA, USA) with 20 µL mixtures containing 10 µL of SYBR Green premix Pro Taq (2×, AG Bio Inc., Hunan, China), 2 µL of soil DNA, 1 µL each of forward and reverse primers, and 6 µL of sterilized distilled water. Melting curve analysis was performed at the end of each PCR run to assess amplification specificity. The standard curve was established through ten‐fold dilution of a standard plasmid containing the target gene, with details provided in Table S4 (Supporting Information), along with the amplification procedure.

### Amplicon Sequencing and Data Processing

The V4‐V5 region of the bacterial 16S rRNA gene was amplified using the primer set 515F/907R. The amplification procedure followed the methods outlined by Liu, et al.^[^ ^78^ ^]^ The PCR products were purified using the AxyPrep DNA Gel Extraction Kit (Axygen Biotechnology, Silicon Valley, CA, USA) and pooled in equimolar concentrations. The sequencing of the PCR products was subsequently conducted at Majorbio Bio‐Pharm Technology Co., Ltd. (Shanghai, China) via the Illumina MiSeq platform (Illumina, San Diego, CA, USA). The 16S rRNA gene sequences have been deposited in NCBI Sequence Read Archive (SRA) database with an accession number PRJNA999205.

The QIIME software (version 1.9.1) was used for processing the raw sequences following the methodology described by Liu, et al.^[^ ^79^ ^]^ Chimeras were filtered out from quality‐controlled and merged sequences using UCHIME.^[^ ^80^ ^]^ The retained sequences were clustered into operational taxonomic units (OTUs) at a 97% similarity threshold. The representative sequence of each OTU was annotated using the Basic Local Alignment Search Tool (BLAST) in the Silva database (Release 138 http://www.arb‐silva.de). Only OTUs with a cumulative count greater than 99 in all the soil samples were retained. The sequences were then rarefied to a total count of 26285 across all the samples.

### Measurement of Root Exudates

The composition of the root exudates was analyzed following a previous study with slight modifications.^[^ ^73^ ^]^ Freeze‐dried root exudates were extracted using 75% methanol. The extraction process involved grinding, sonicating, and centrifuging. The volume of the extracted exudates dissolved in the methanol solution relative to the fresh weight of the roots was analyzed using an ultrahigh‐performance liquid chromatograph coupled with a Fourier transform mass spectrometer (UHPLC‐Q Exactive HF‐X system, Thermo Scientific, Waltham, MA, USA). UHPLC analysis was performed using an ACQUITY UPLC HSS T3 chromatographic column (100 mm × 2.1 mm, 1.8 µm, Waters, Milford, CT, USA). To ensure the stability of the testing process, a quality control sample was prepared by combining 20 µL of supernatant from each sample, and it was injected every 5–10 samples during detection. The raw data were analyzed using Progenesis QI software (Waters, Milford, MA, USA), and compound identification was conducted using the KEGG database (https://www.kegg.jp) and HMDB (http://www.hmdb.ca). Compounds with a relative standard deviation of less than 30% in the quality control sample were selected for further analysis.

### Metagenomic Sequencing

Metagenomic sequencing was performed via paired‐end sequencing on the Illumina HiSeq 2500 platform (Magigene Co., Ltd., Shenzhen, Guangdong, China). The raw reads of metagenomic sequencing were deposited in the NCBI SRA database under accession number PRJNA1188331. Trimmomatic software was used to filter out low‐quality data, resulting in more than 8 Gb of clean data per sample. The clean data were assembled using the Megahit program, and subsequently, open reading frames were predicted. The nonredundant gene catalog was constructed using the LINCLUST program. The relative abundance of each gene was quantified as transcripts per million (TPM) using the BBMAP program. ARGs and MGEs were annotated using the Comprehensive Antibiotic Resistance Database (CARD) and MobileOG‐db database, respectively. The ARG‐carrying contigs were BLAST searched against the NR database of the NCBI to identify potential hosts. ARG hosts that are bacterial pathogens were also identified from the A‐to‐Z database.^[^ ^81^ ^]^ ARG risk levels were classified by a framework proposed by Zhang, et al.^[^ ^6^ ^]^

### Bioinformatics and Statistical Analysis

Most analyses were conducted using R software (version 4.2.0). Principal coordinate analysis (PCoA) was performed with the “*ordinate*” function in the “*phyloseq*” package to compare differences in the ARG and MGE profiles based on the basis of the Bray‐Curtis matrix.^[^ ^82^ ^]^ PERMANOVA was applied to test the effects of plant diversity and species on ARG and MGE abundance using the “*adonis2*” function in the “*vegan*” package after permutational analysis of multivariate dispersions.^[^ ^83^ ^]^ Correlation analysis has largely been used for the identification of ARG hosts.^[^ ^76^, ^84^, ^85^, ^86^, ^87^ ^]^ Here, Spearman rank correlation analysis with the “*rcorr*” function in the “*Hmisc*” package was used to identify potential ARG and MGE hosts. OTUs exhibiting a significant positive correlation (*ρ* > 0.6 and *p* < 0.01) with ARGs and MGEs were considered potential hosts. The pathogenicity of these potential hosts was identified via a multiple bacterial pathogen detection pipeline as described by Yang, et al.^[^ ^88^ ^]^ Microbial α diversity indices were calculated with the “*vegan*” package. Constrained analysis of principal coordinates (CAP) was conducted using the “*ordinate*” function in the “*phyloseq*” package to examine the impact of plant diversity on microbial community structure. Random forest analysis was performed to identify key bacterial OTUs that respond to changes in plant diversity using the “*rfPermute*” package. Spearman correlation analysis was used to analyze cooccurrence relationships among bacterial OTUs, with relationships meeting the criteria of |*ρ*| > 0.8 and *p* < 0.01 retained and visualized with Gephi software (version 0.9.2). PCoA was performed to examine the impact of plant species on microbial community structure. The chemical diversity indices of the root exudates were calculated via the “*vegan*” package. The impact of plant diversity on root exudate profiles was analyzed using CAP, PERMANOVA, and variance partitioning analysis (VPA). The impact of the plant pattern on the root exudate profile was analyzed with PCoA, PERMANOVA, and VPA. Spearman correlation was used to calculate the correlations among plant root exudate compounds, with associations of |*ρ*| > 0.7 and *p* < 0.01 retained and visualized via Cytoscape 3.10.0 software. The neighborhood connectivity index of each compound in the co‐occurrence network of plant root exudates was calculated via Cytoscape 3.10.0 software. Random forest analysis was performed to identify key root exudates that respond to changes in plant diversity and species. The impact of plant species on the root exudate profiles was analyzed via PCoA, PERMANOVA, and VPA as described above. Associations among plant root exudate compounds and between potential hosts and plant root exudates were analyzed using Spearman correlation, with associations of |*ρ*| > 0.7 and *p* < 0.01 retained and visualized. SmartPLS 4 software was used for partial least square‐structural equation modeling to examine the impact of plant diversity on reducing ARG and MGE abundance. The impacts of resource diversity on ARG abundance and composition were assessed with CAP and PERMANOVA, as described earlier. Random forest analysis and PERMANOVA were used to determine the effects of resource diversity and specific resources on soil ARG abundance. The impact of resource diversity on microbial communities was analyzed using CAP and PERMANOVA. Network analysis of ARGs, MGEs and pathogenic bacteria was performed via Cytoscape software. One‐ and two‐ way analysis of variance (ANOVA) and regression analysis were performed using SPSS software (version 20, SPSS Inc., Chicago, IL, USA).

### Ethical Statement

This study did not involve human or animal subjects, and thus, no ethical approval was required. The study protocol adhered to the guidelines established by the journal.

## Conflict of Interest

The authors declare no conflict of interest.

## Author Contributions

S.L. designed the study, performed the experiments, analyzed the data, and wrote the paper. X.Z. and L.L. designed the study and performed the experiments. S.Z. performed the experiments. J.Z. and J.Z. revised the paper. Z.C. conceived the study, provided resources, and revised the paper. J.P. wrote and revised the paper. X.H. conceived and designed the study, participated in analyzing the data and writing the paper, and contributed resources. All authors read and approved the version to be published.

## Supporting information



Supporting Information

## Data Availability

The data that support the findings of this study are available from the corresponding author upon reasonable request.
