# Supplementary material for: Plant Diversity Reduces the Risk of Antibiotic Resistance Genes in Agroecosystems
Source: Adv Sci (Weinh). 2025 Jan 28;12(11):2410990. doi: 10.1002/advs.202410990 (PMC11923964; doi:10.1002/advs.202410990)
Supplement: Supplementary file 1 — Supporting Information [file ADVS-12-2410990-s001.docx]

Supporting Information

Plant Diversity Reduces the Risk of Antibiotic Resistance Genes in Agroecosystems

*Shu Li, Xing Zhou, Liangliang Liu, Zhe Su, Jun Zhao, Jinbo Zhang, Zucong Cai, Josep Peñuelas, Xinqi Huang*

**
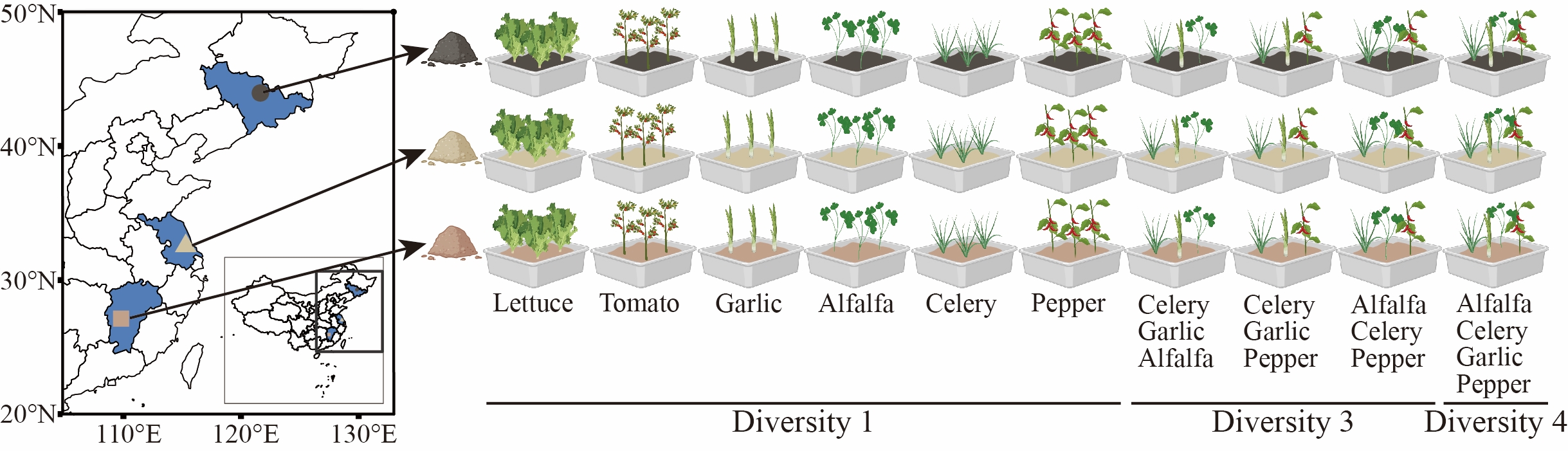
**

**Figure S1. Schematic diagram of the experimental design.** Plants with different species and diversities were planted in the black soil (BS), fluvo-aquic soil (FS), and red soil (RS) for two consecutive seasons within seven months. The soil samples were analyzed after plant cultivation.


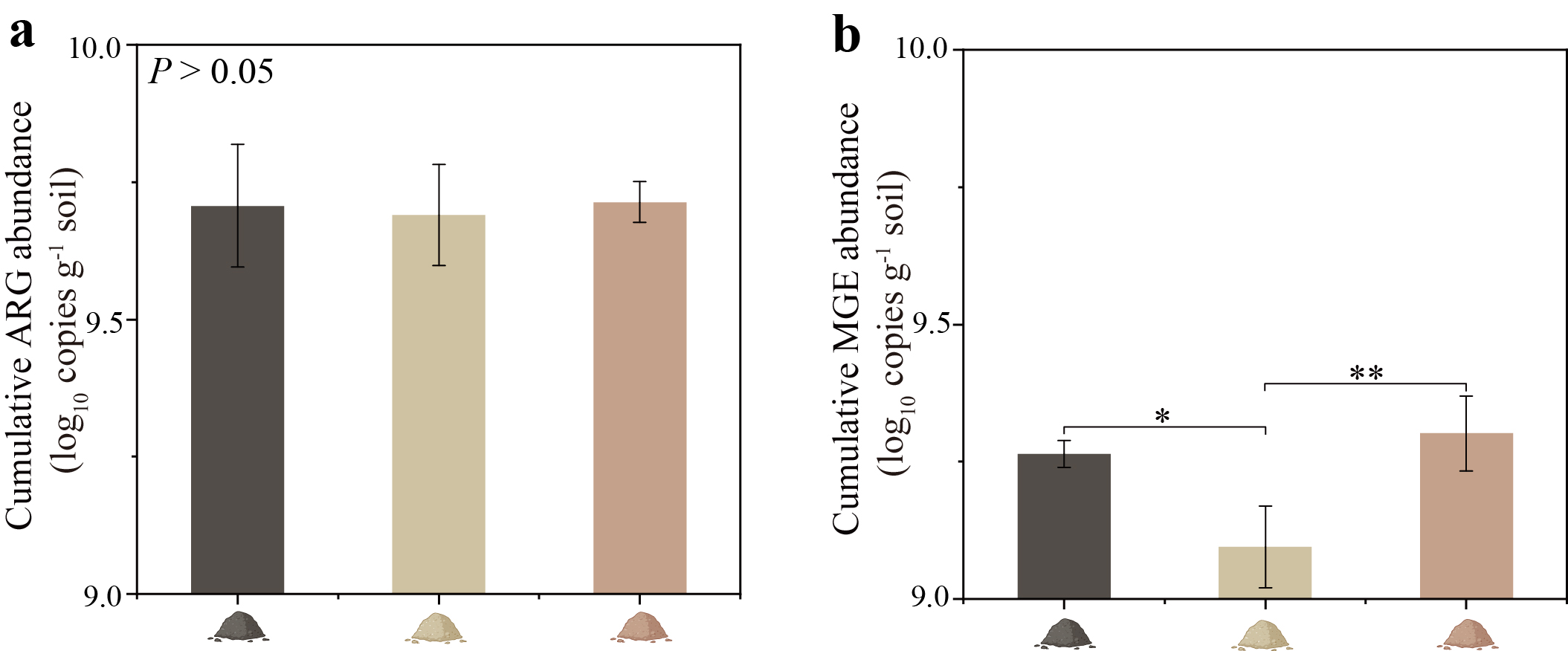


**Figure S2. Initial cumulative abundance of antibiotic resistance genes (ARGs, a) and mobile genetic elements (MGEs, b) in three soil types.** The symbols * and ** denote *P* < 0.05 and 0.01 according to one-way ANOVA and Duncan’s test, respectively.


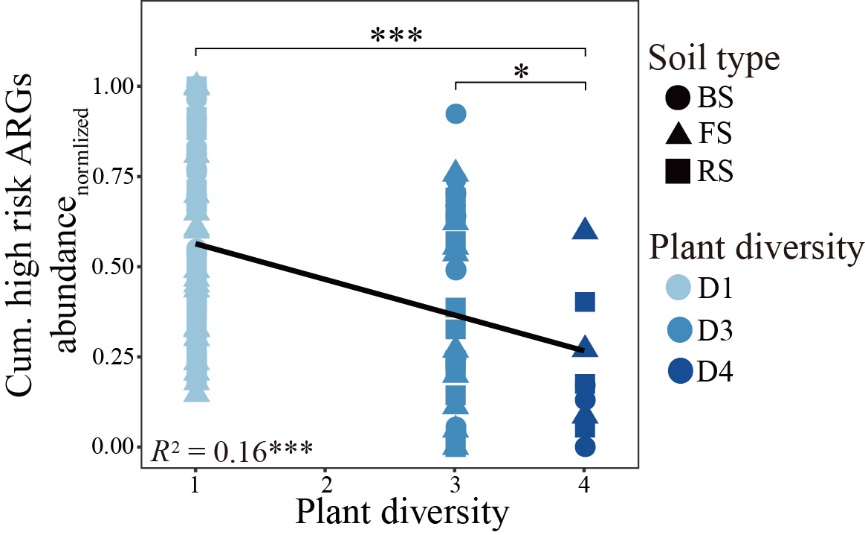


**Figure S3. Response of cumulative abundance of the high-risk antibiotic resistance genes (ARGs) and mobile genetic elements (MGEs) to plant diversity in each soil type.** The data were max-min normalized within each soil type. The *R^2^* and *P*-value were determined by the general linear model and the significance was detected by one-way ANOVA and Duncan’s test. The symbols * and *** denote *P* < 0.05 and 0.001, respectively.


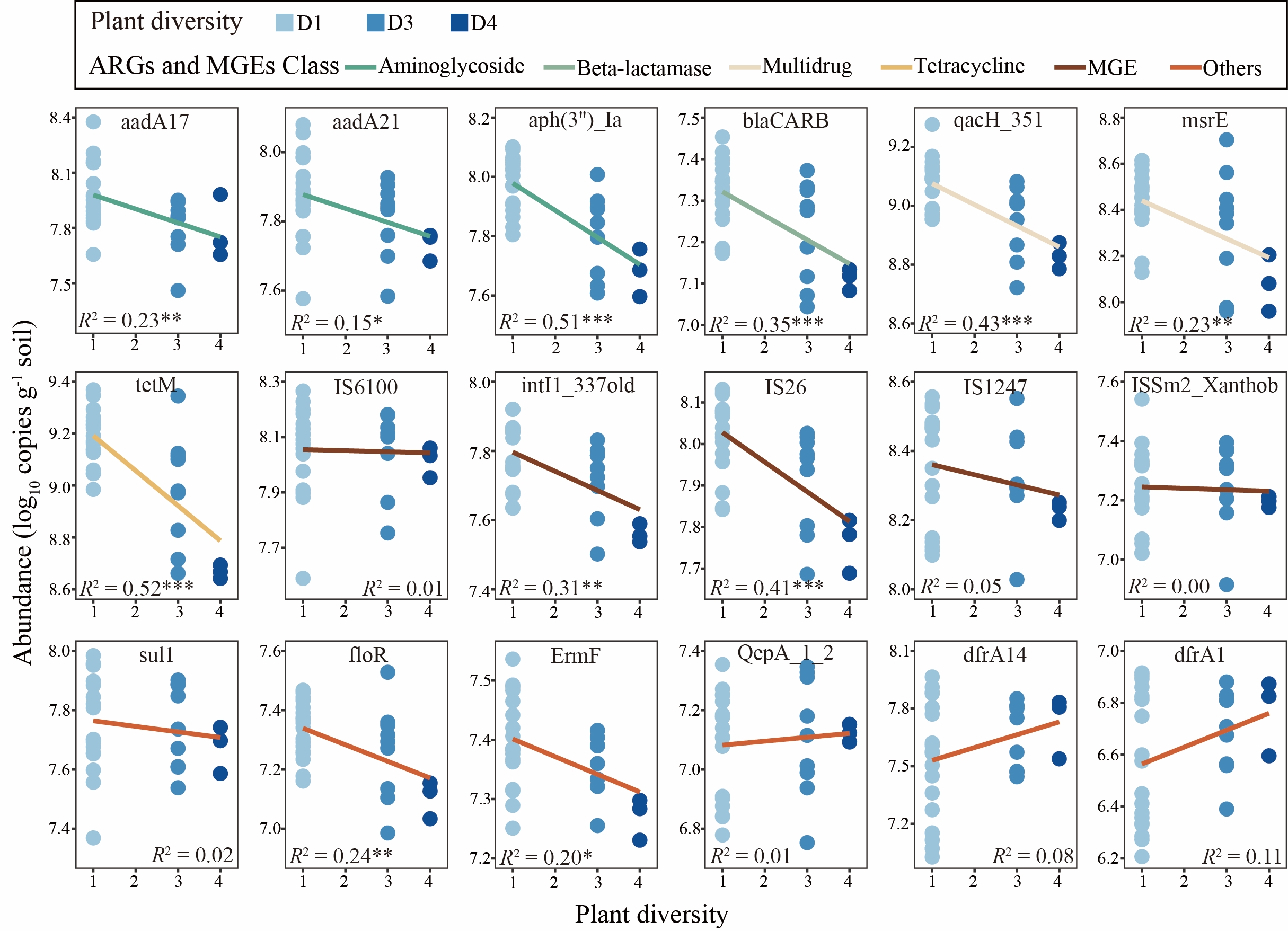


**Figure S4. Responses of the 18 abundant antibiotic resistance genes (ARGs) and mobile genetic elements (MGEs) to plant diversity in the BS.** The *R^2^* and *P*-value were determined by the general linear model and the symbols *, ** and *** denote *P* < 0.05, < 0.01, and < 0.001, respectively.


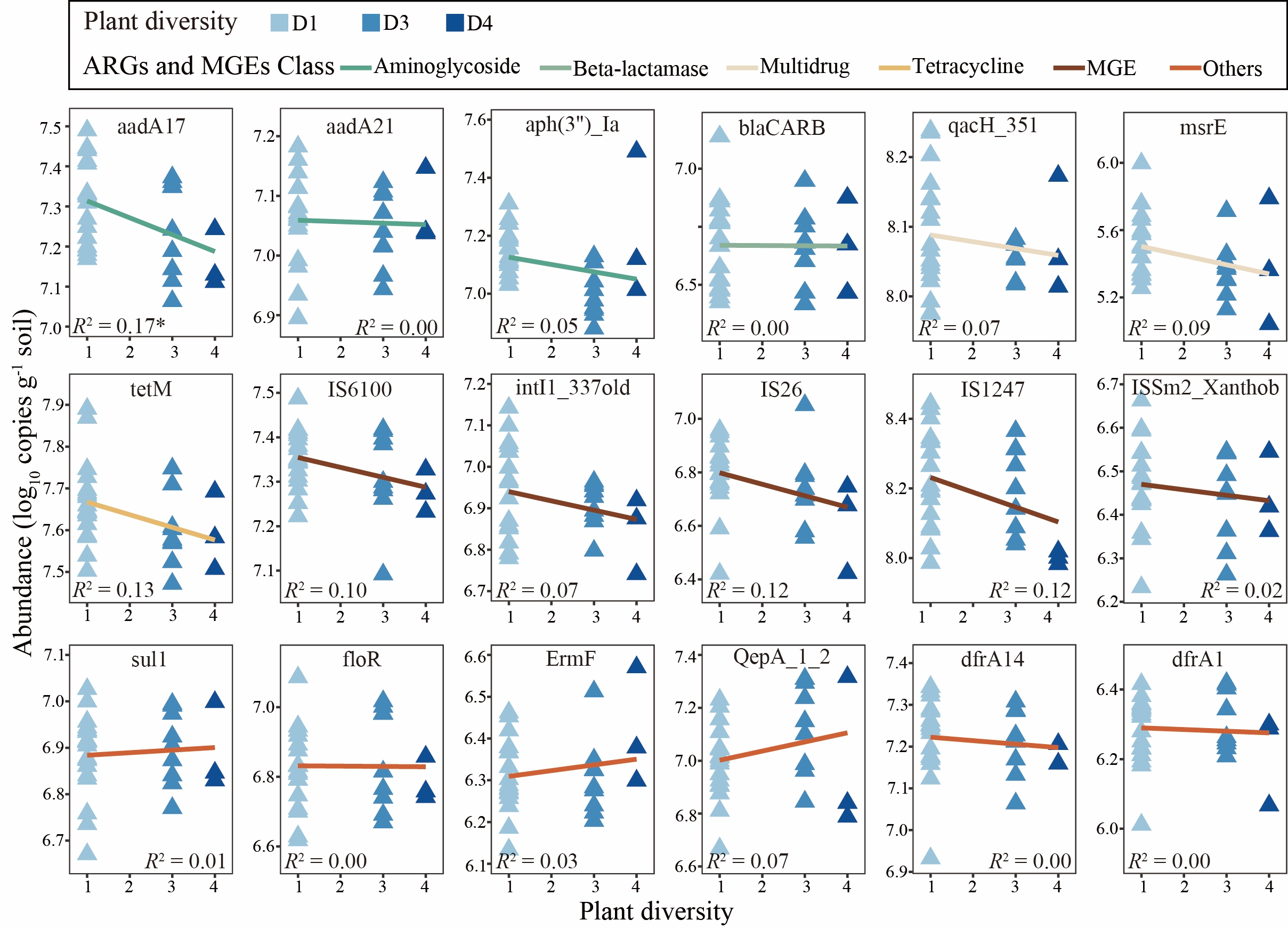


**Figure S5.** **Responses of the 18 abundant antibiotic resistance genes (ARGs) and mobile genetic elements (MGEs) to plant diversity in the FS.** The *R^2^* and *P*-value were determined by the general linear model and the symbol * denotes *P* < 0.05.


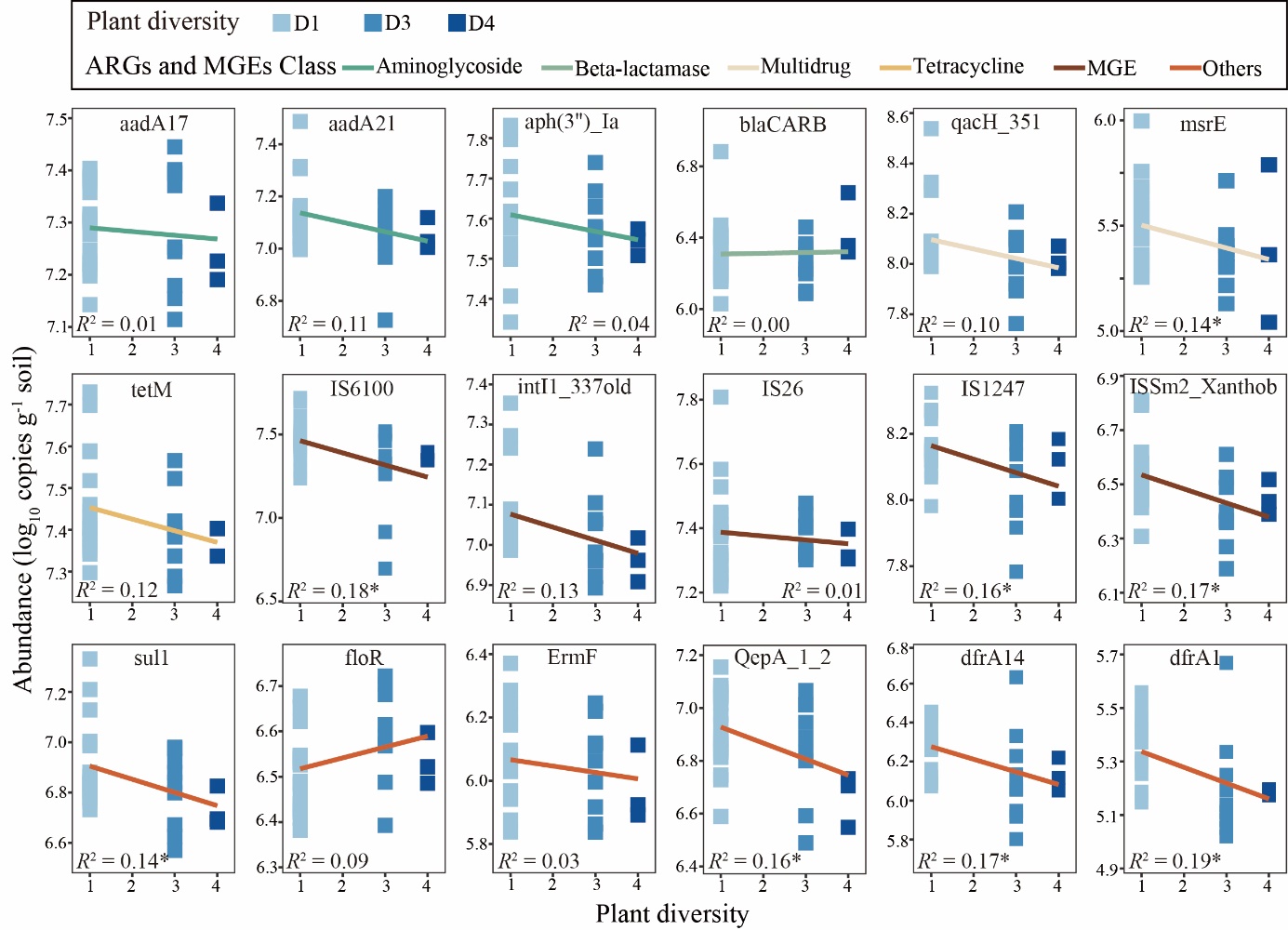


**Figure S6.** **Responses of the 18 abundant antibiotic resistance genes (ARGs) and mobile genetic elements (MGEs) to plant diversity in the RS**. The *R^2^* and *P*-value were determined by the general linear model and the symbol * denotes *P* < 0.05.


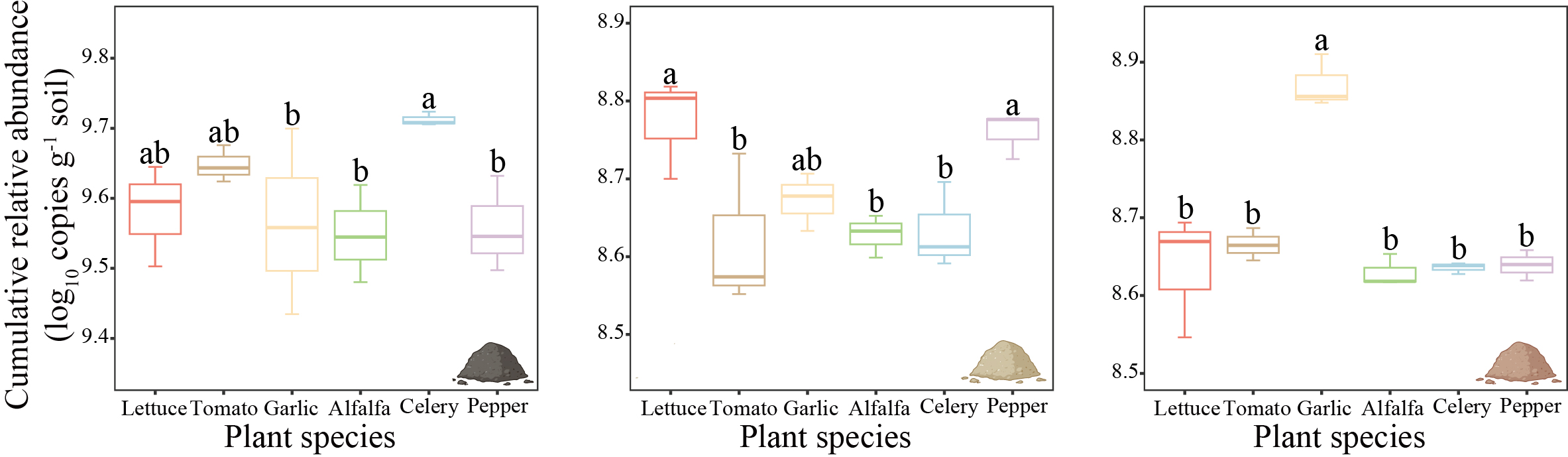


**Figure S7. Responses of the cumulative relative abundance of 18 abundant antibiotic resistance genes (ARGs) and mobile genetic elements (MGEs) to plant species in the three soils.** Different letters represent significant differences at *P* < 0.05 according to one-way ANOVA and Duncan’s test.


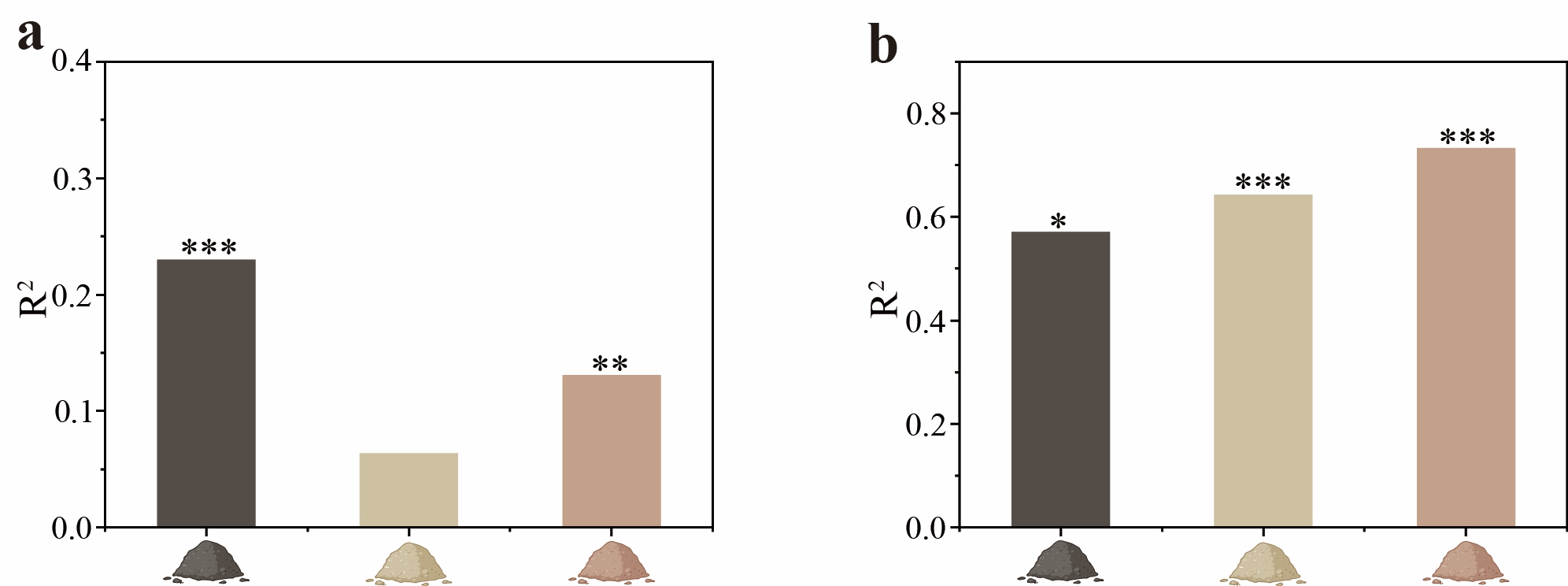


**Figure S8. Deterministic effects (*R^2^*) of plant diversity (a) and species (b) on antibiotic resistance gene (ARGs) and mobile genetic elements (MGEs) composition in the three soils**. The *R^2^* and *P-*value were determined by PERMANOVA. The symbols *, **, and *** denote *P* < 0.05, < 0.01, and < 0.001, respectively.


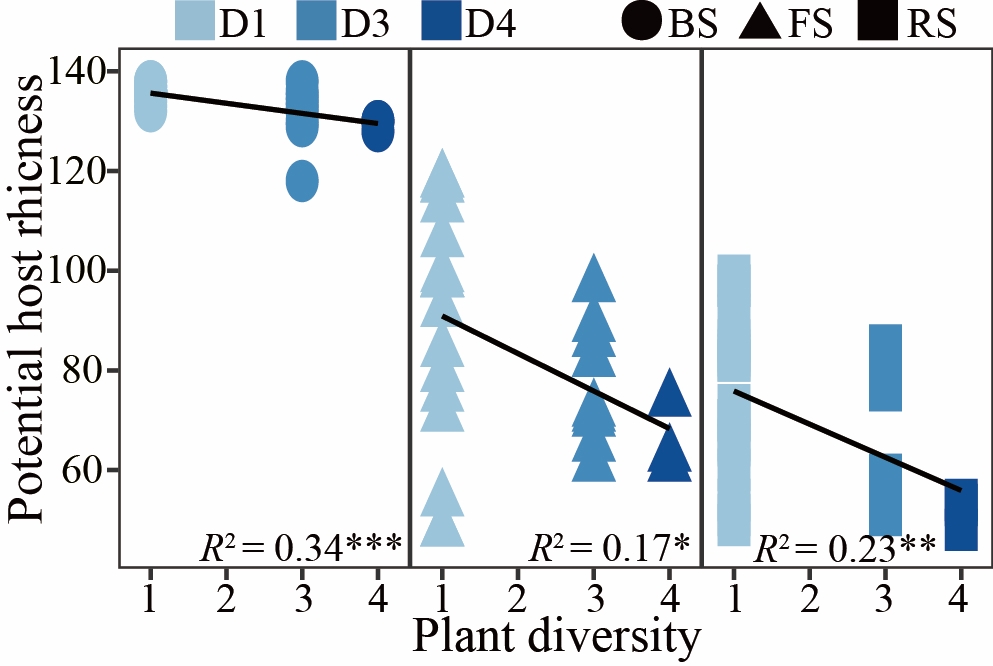


**Figure S9. Responses of the richness of potential hosts to plant diversity.** The *R^2^* and *P*-value were determined by the general linear model and the symbols *, **, and *** denote *P* < 0.05, < 0.01, and < 0.001, respectively.


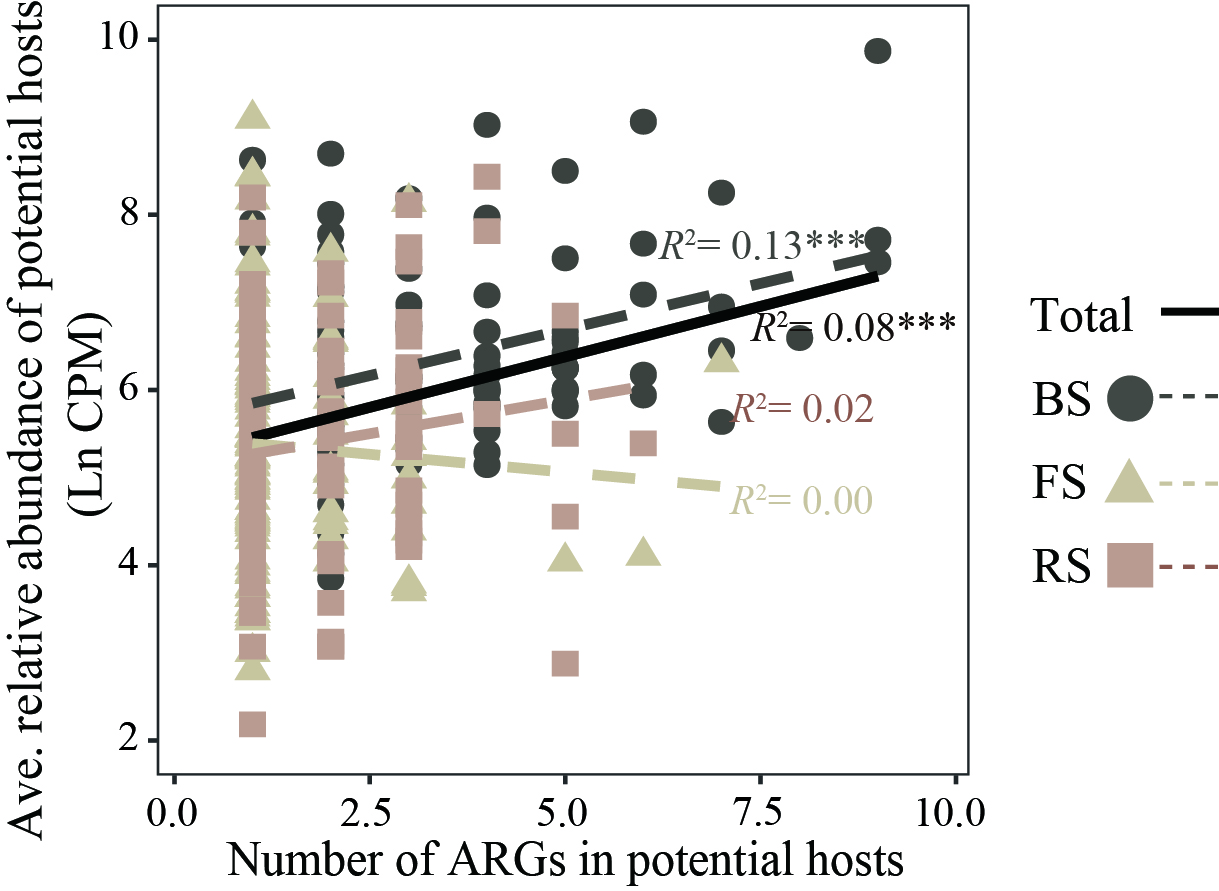


**Figure S10.** **Relationships between the number of antibiotic resistance genes (ARGs) in potential hosts and their average relative abundance.** The *R^2^* and *P*-value were determined by the general linear model and the symbol *** denotes *P* < 0.001.


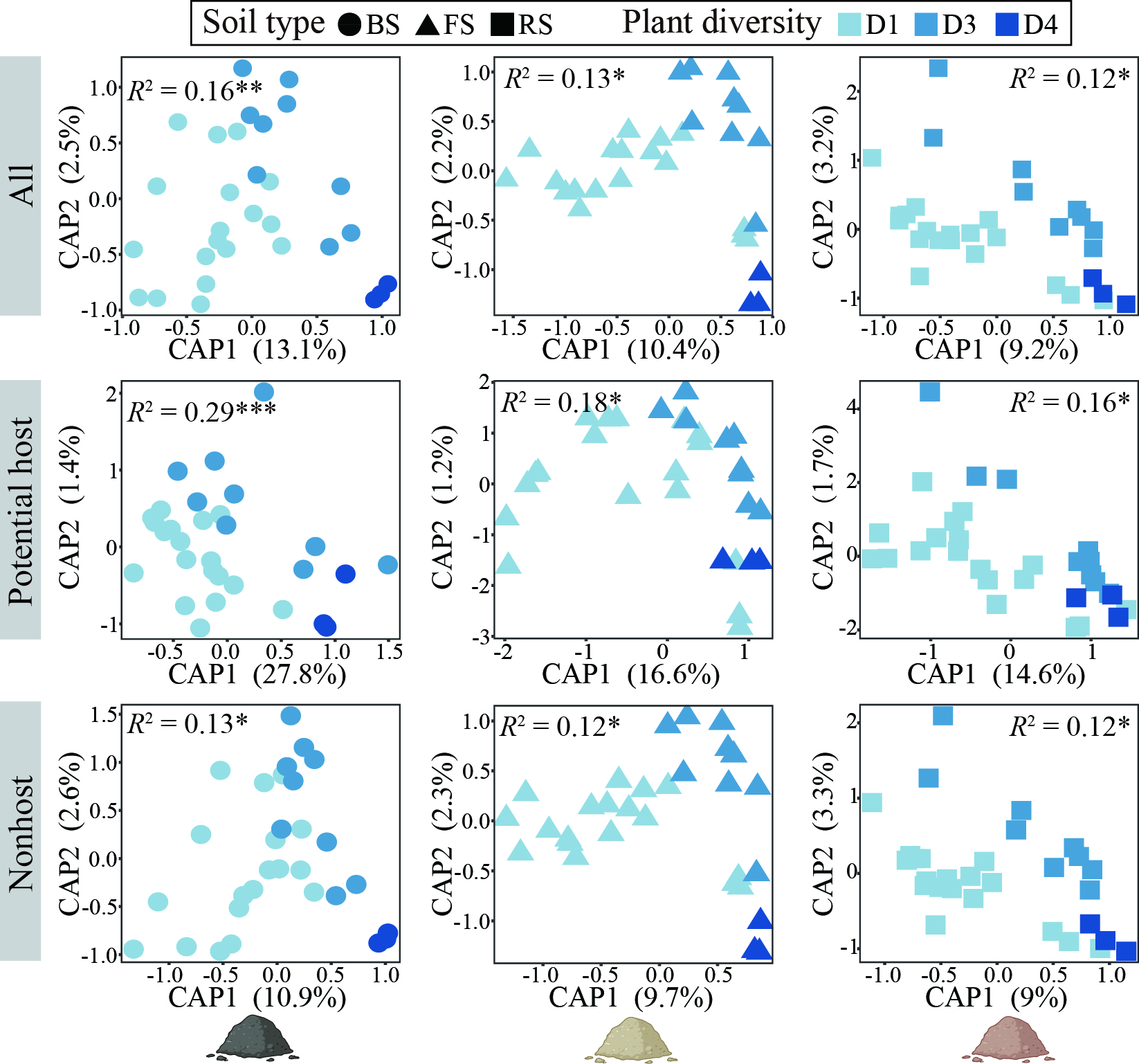


**Figure S11.** **Constrained analysis of the principal coordinates of the bacterial OTUs affiliated with different groups under different plant diversities.** The *R^2^* and *P-*value were determined by PERMANOVA and the symbols *, **, and *** denote *P* < 0.05, < 0.01, and < 0.001, respectively.


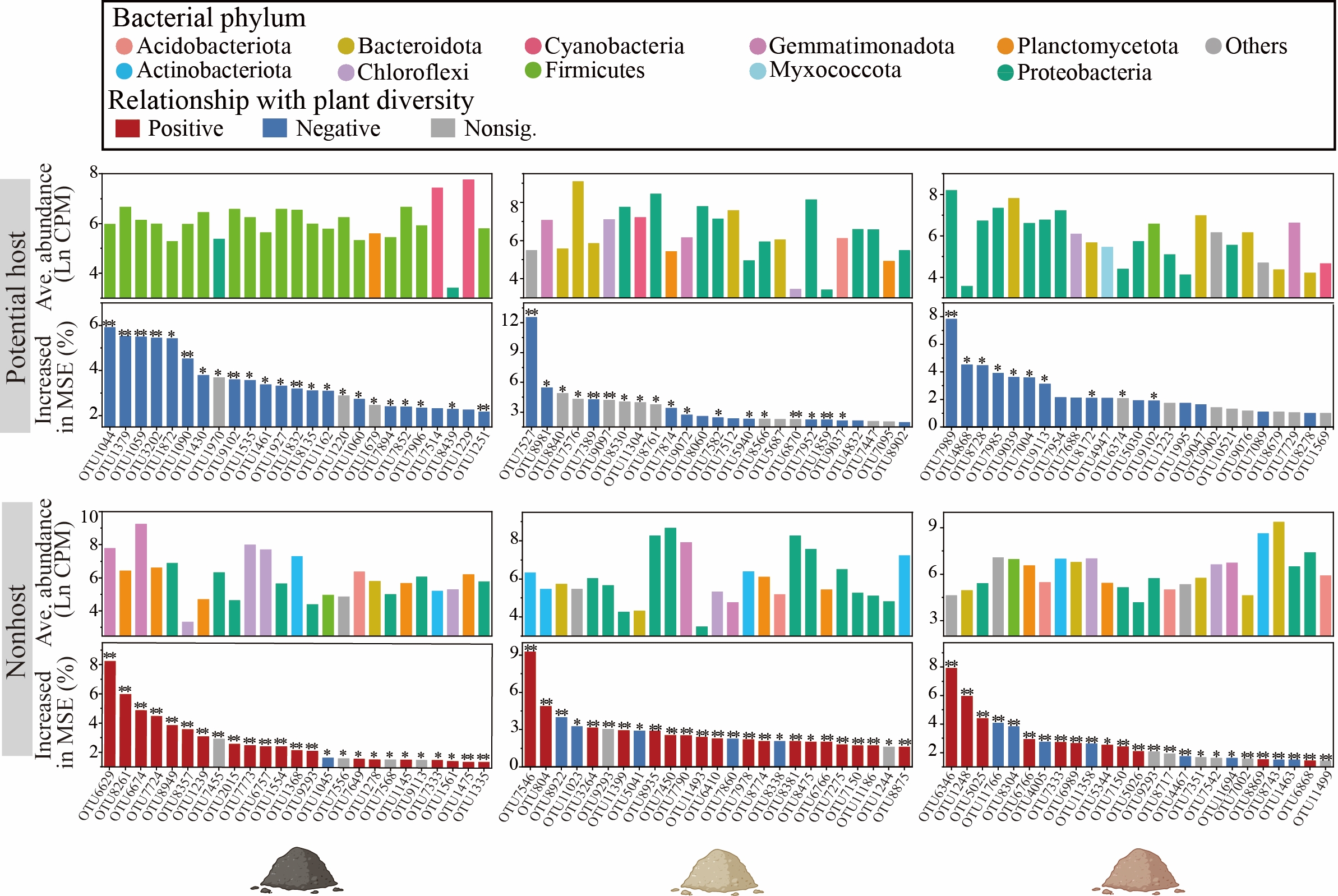


**Figure S12.** **Random forest analysis showing the key OTUs related to plant diversity.** The upper plots show the average relative abundance of these key OTUs, and the color of the bar indicates its taxonomic classification in phylum level. The lower plots show the increased in mean squared error (MSE) of these OTUs determined by random forest analysis, and the color of the bar indicates its relationship, based on Pearson correlation analysis, with plant diversity. The symbols *, and ** denote *P* < 0.05, and <0.01, determined by random forest analysis, respectively.


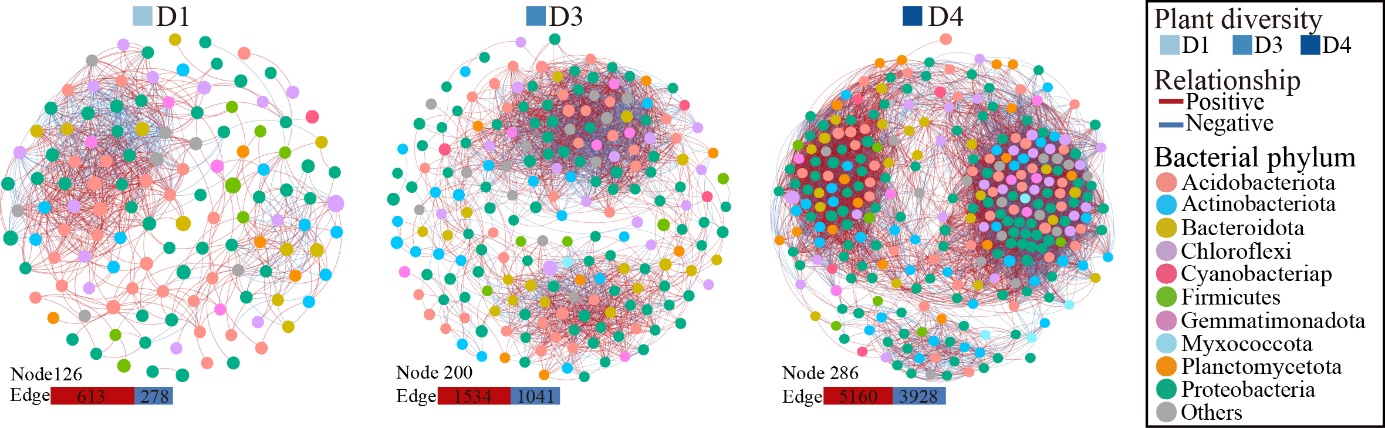


**Figure S13.** **Network analysis of the bacterial interactions under different plant diversities.** Significant (*P* < 0.01) and close (|*ρ*| > 0.8) relationships based on Spearman rank correlation analysis are retained as edges.


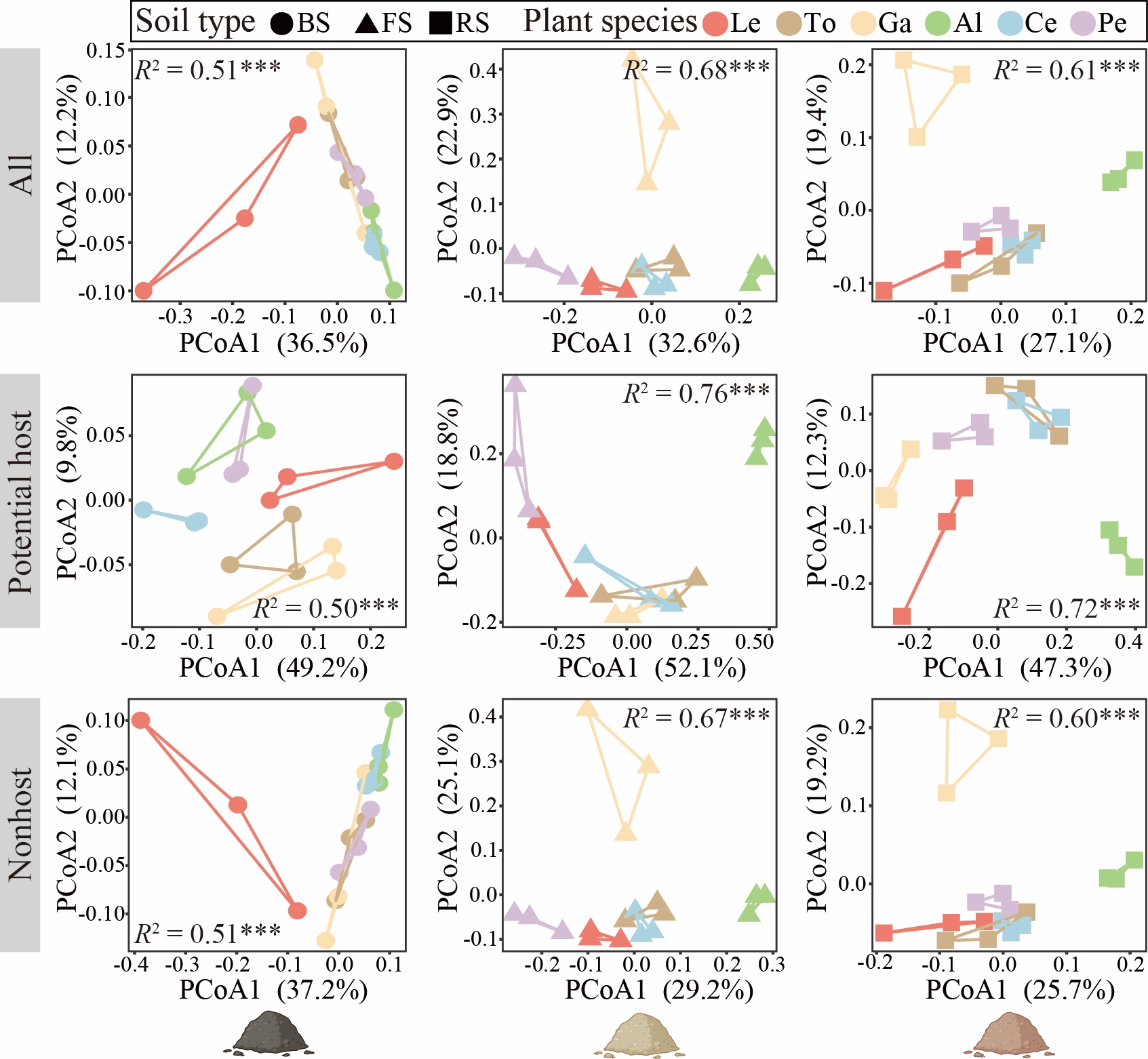


**Figure S14.** **Principal coordinate analysis of the bacterial OTUs in the soils cultivated with different plant species.** The *R^2^* and *P-*value were determined by PERMANOVA and the symbol *** denotes *P* < 0.001.


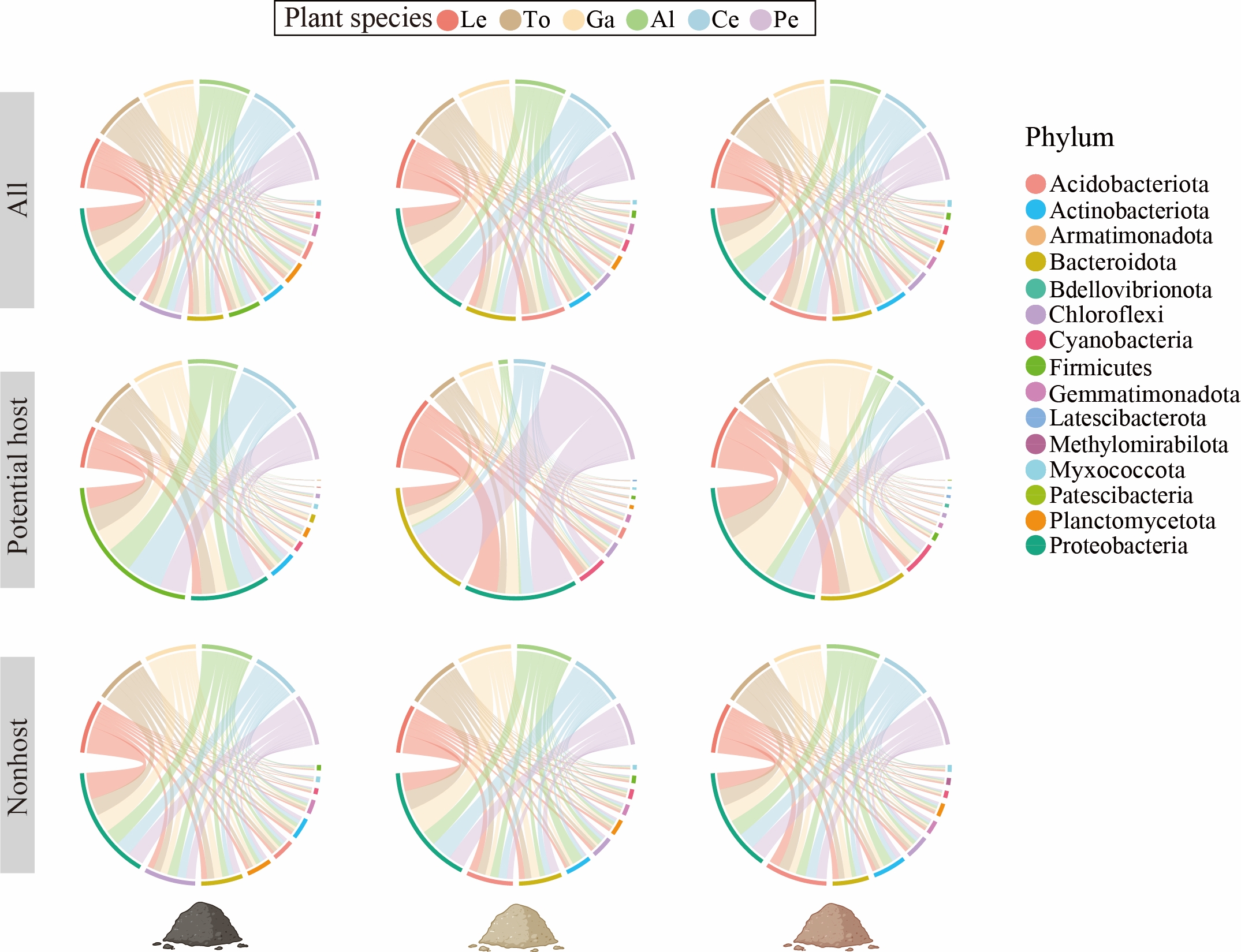


**Figure S15. Bacterial composition in the soils cultivated with different plant species.**

**
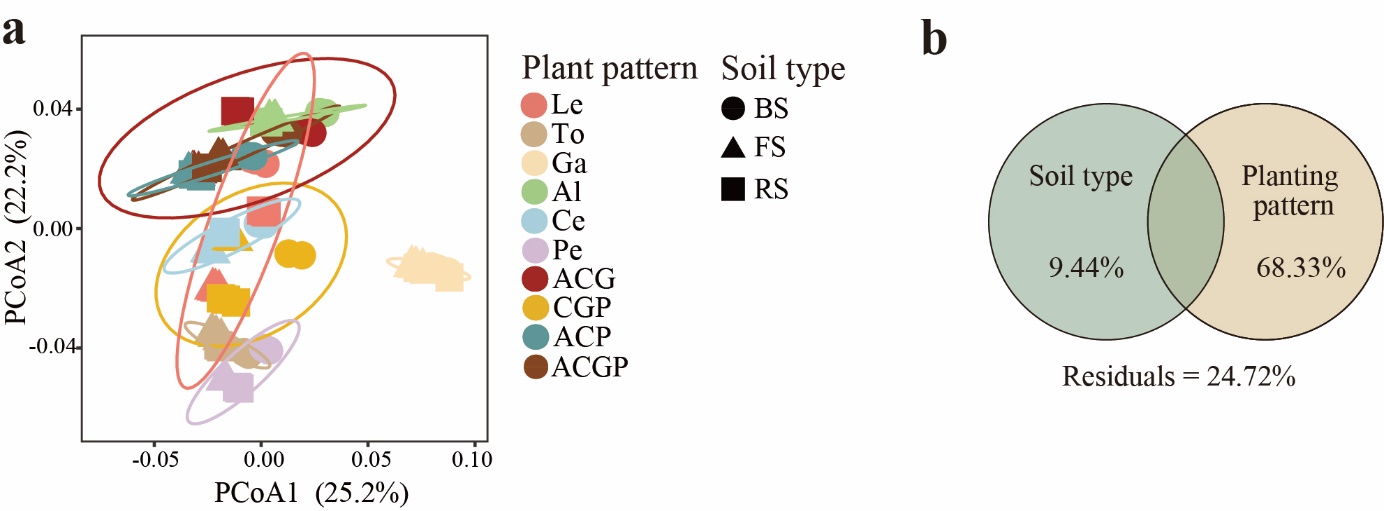
**

**Figure S16. Root exudate profiles under different planting patterns in the three soils. a,** Principal coordinate analysis of root exudate profiles. **b,** Variance partitioning analysis of the effects of planting pattern and soil type on root exudate profiles.

**
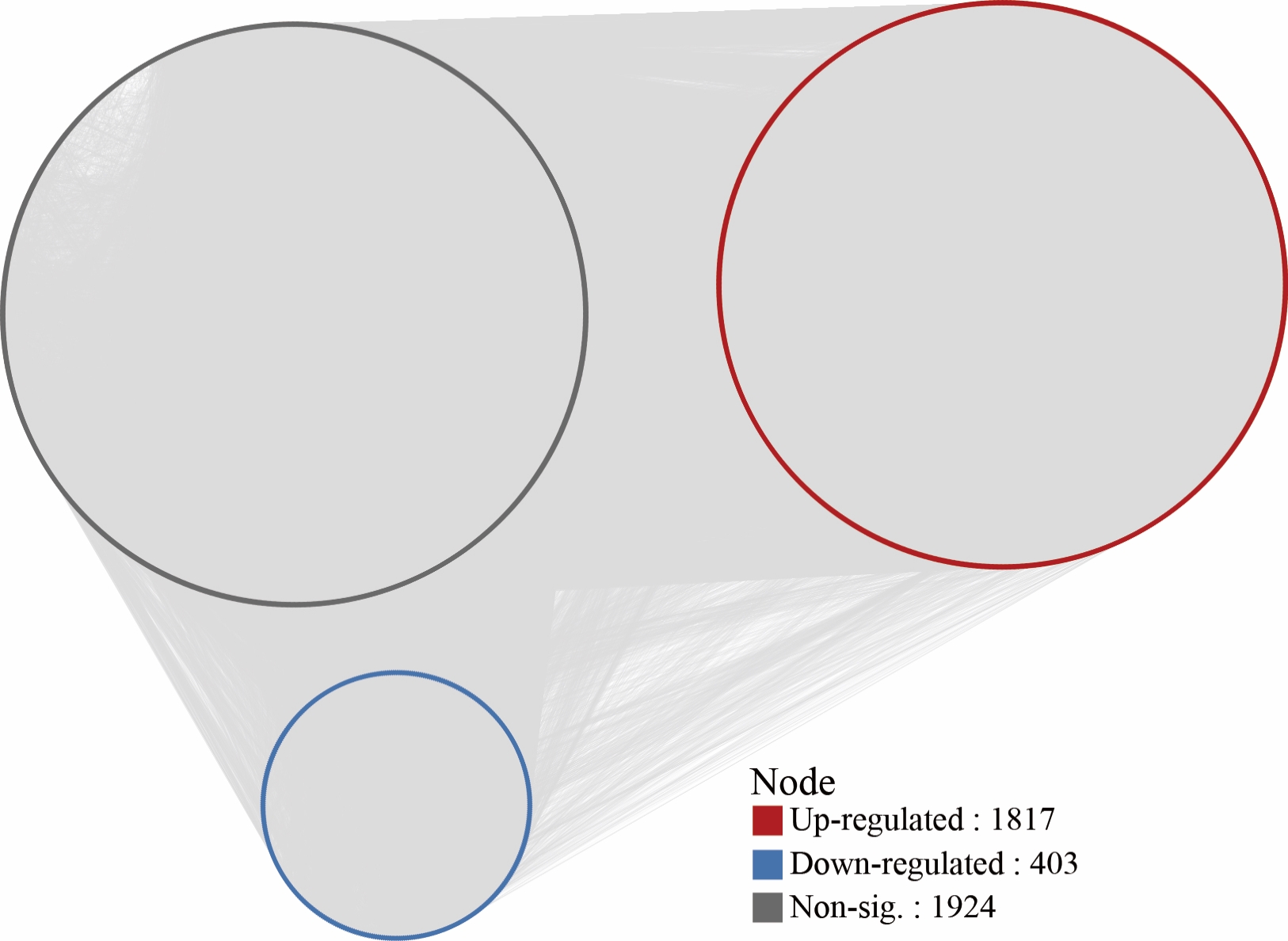
**

**Figure S17. Network analysis of the compounds in root exudates.** Significant (*P* < 0.01) and close (|*ρ*| > 0.7) relationships based on Spearman rank correlation analysis are retained as edges.


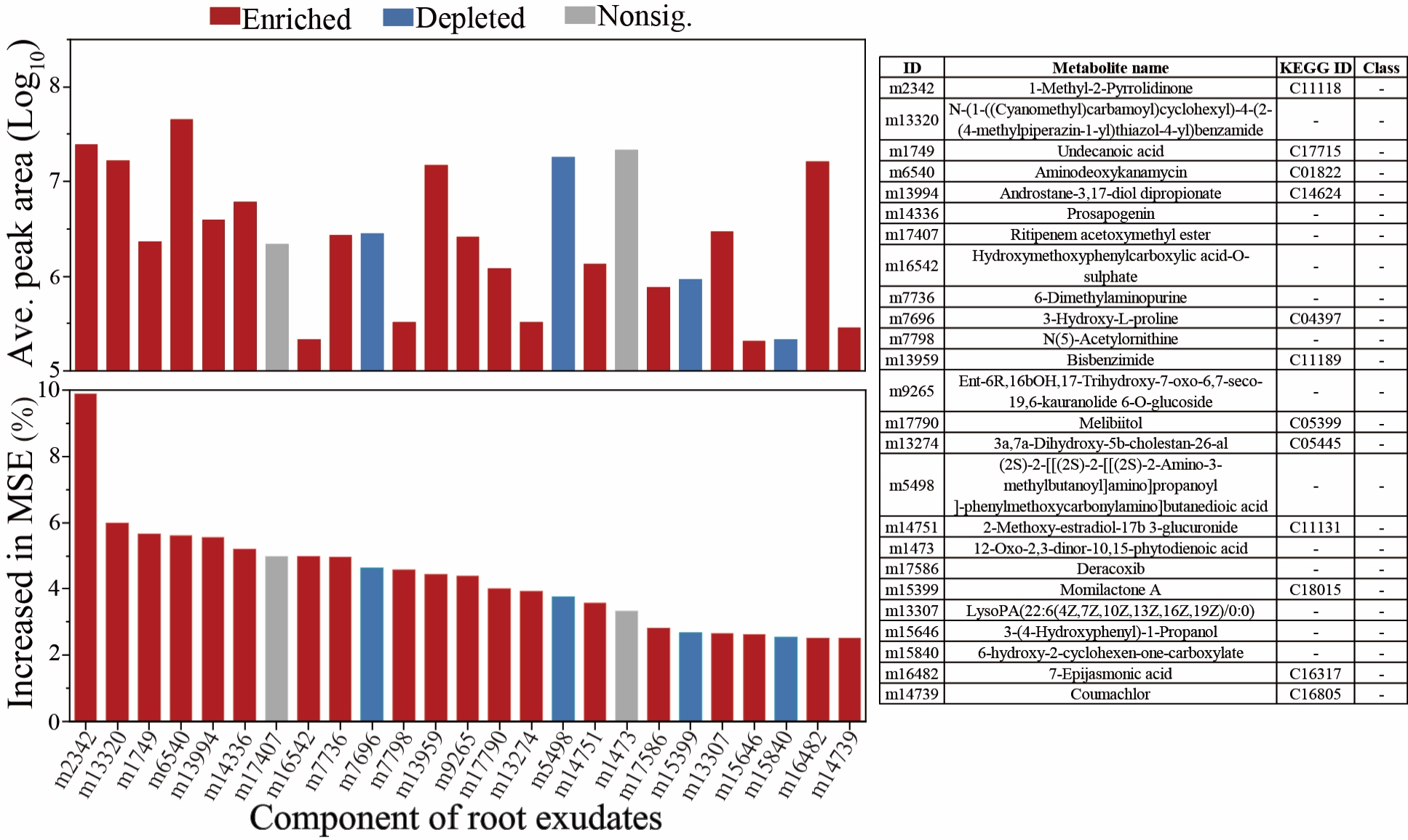


**Figure S18.** **Key root exudate components among different plant diversities and their average peak areas**. The color of the bar indicates its relationship with plant diversity detected by Pearson correlation analysis. The symbol “-” in the column of “Class” represents unclassified compounds.

**
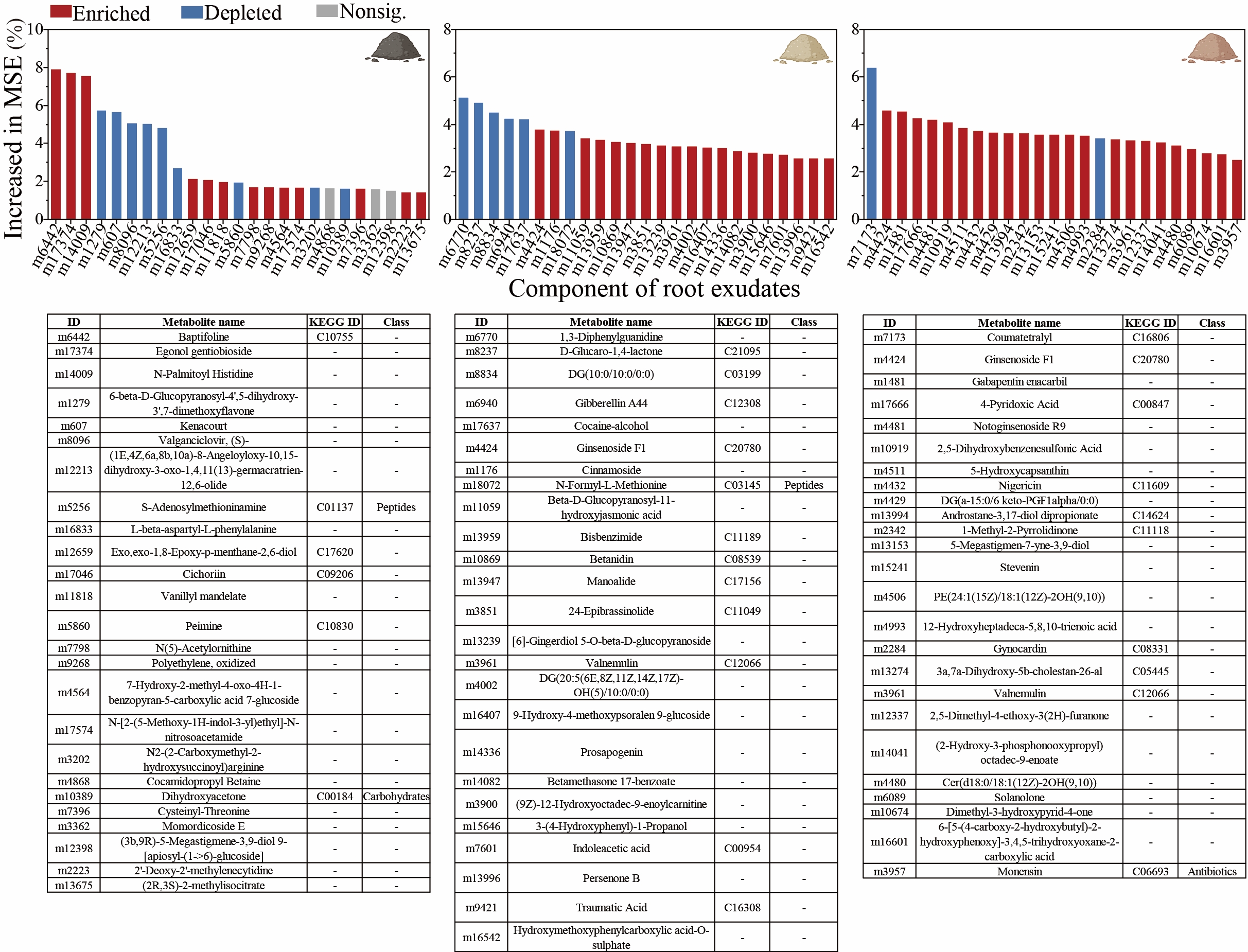
**

**Figure S19. Key root exudate components among the different plant diversities in each soil.** The color of the bar indicates its relationship with plant diversity detected by Pearson correlation analysis. The symbol “-” in the column of “Class” represents unclassified compounds.


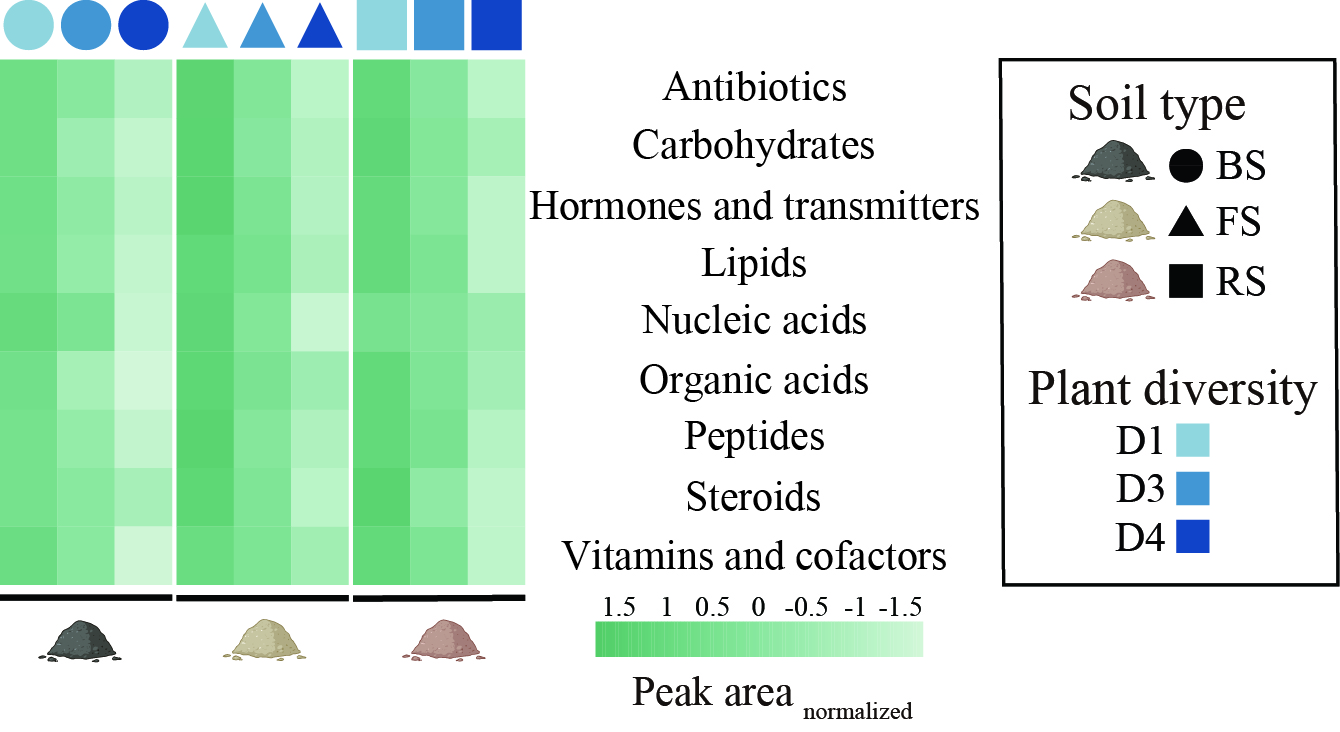


**Figure S20. Relationships between the well-classified compounds and plant diversity in each soil.** The heatmap indicates the peak area of each class (cumulative peak area of all compounds in this class) in different plant diversity gradients.


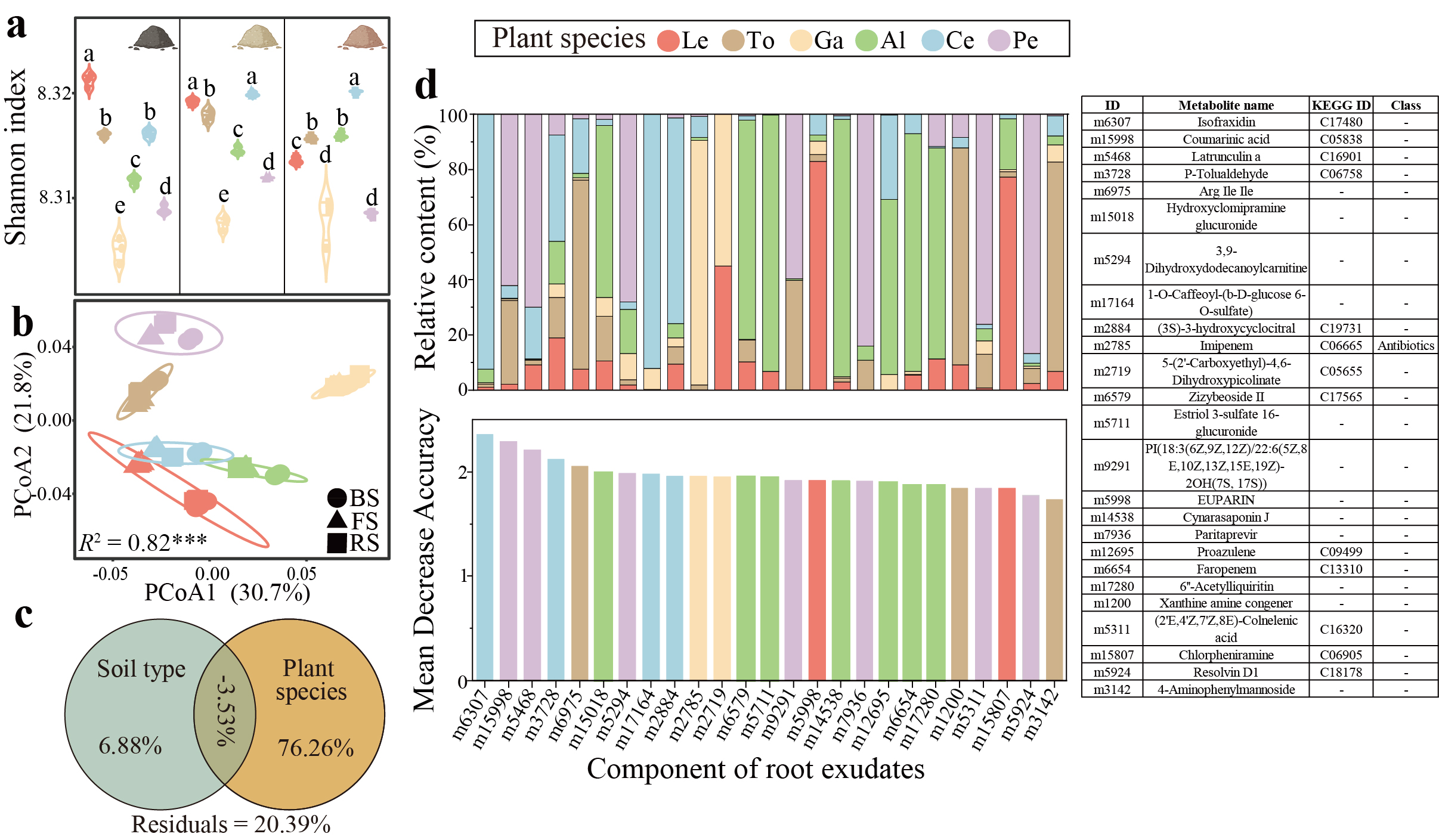


**Figure S21.** **Influence of plant species on root exudates.** **a,** Shannon diversity index of root exudates. Different letters represent significant differences at *P* < 0.05 according to one-way ANOVA and Duncan’s test. **b,** Principal coordinate analysis of root exudate profiles. The *R^2^* and *P*-value were determined by PERMANOVA. **c,** Variation partitioning analysis showing the contributions of soil type and plant species to the variation of root exudates. **d,** Key root exudate components among different plant species identified by random forest analysis and their relative contents. The symbol “-” in the column of “Class” represents unclassified compounds. The symbol *** denotes *P* < 0.001.

**
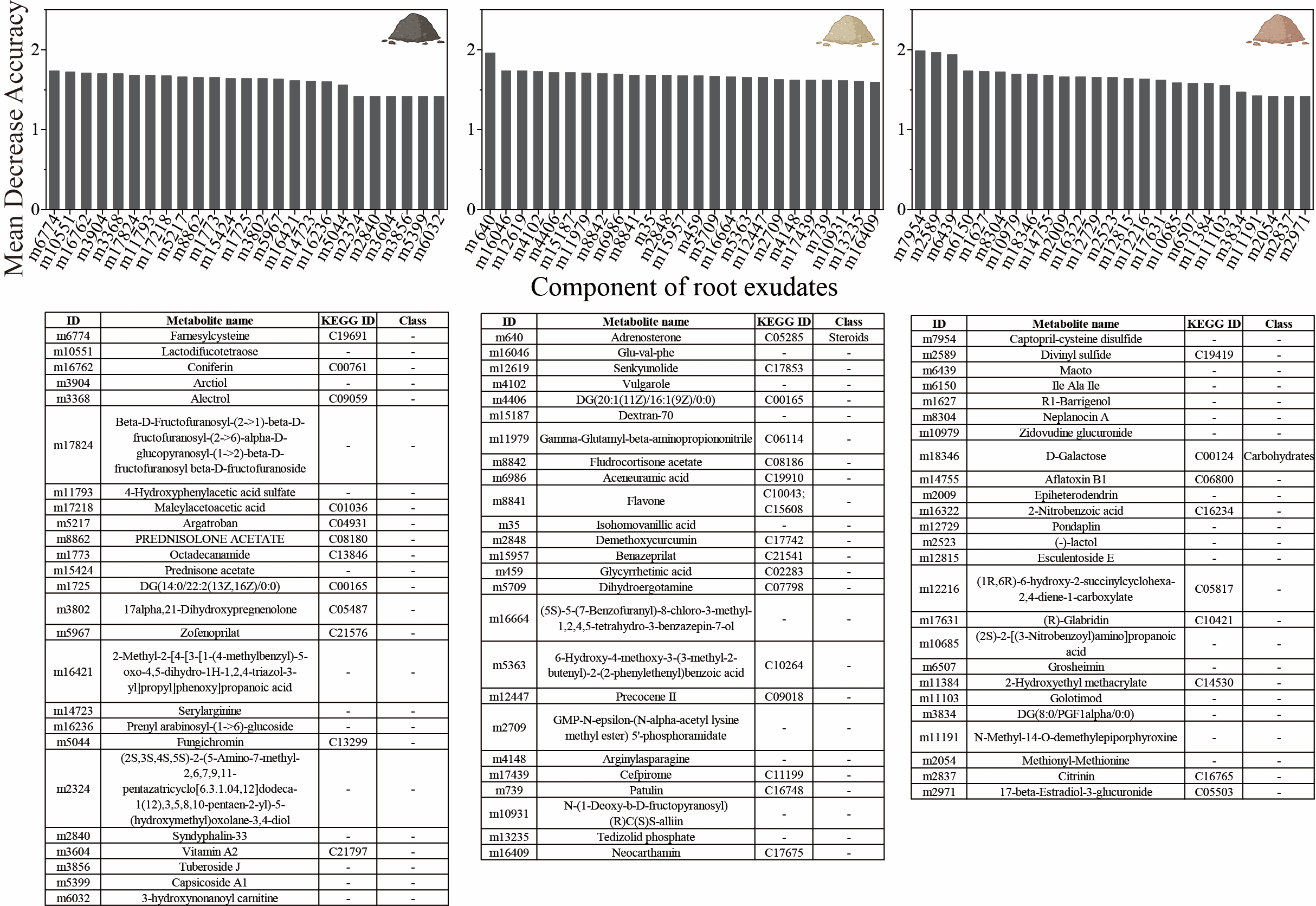
**

**Figure S22. Key root exudate components among different plant species in each soil.** The symbol “-” in the column of “Class” represents unclassified compounds.


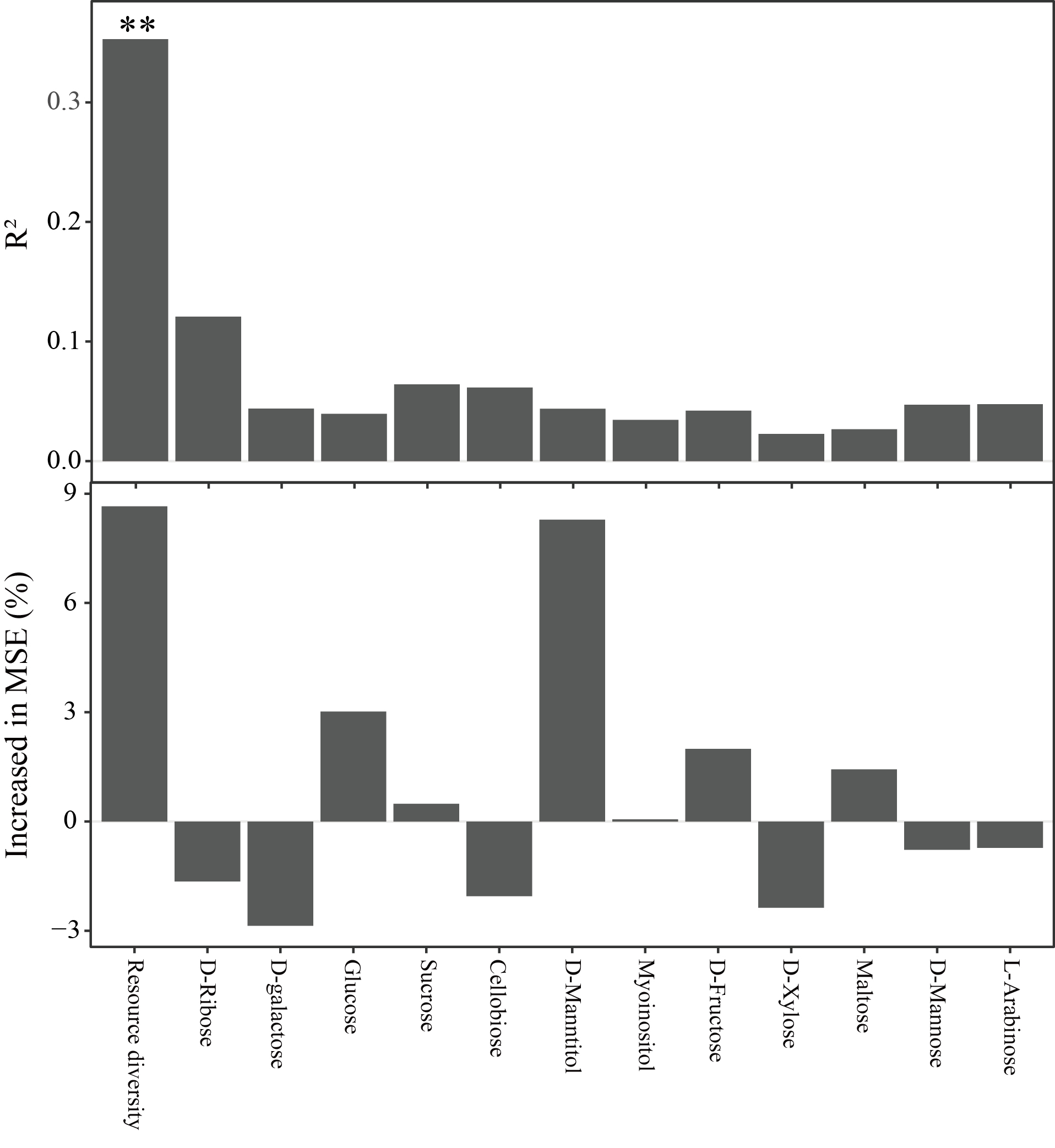


**Figure S23. Effects of resource diversity and specific resources on antibiotic resistance gene (ARG) composition (upper) and total abundance (lower).** The effects on ARG composition and total abundance were calculated by PERMANOVA and random forest analysis, respectively. The symbol ** denotes *P* < 0.01.


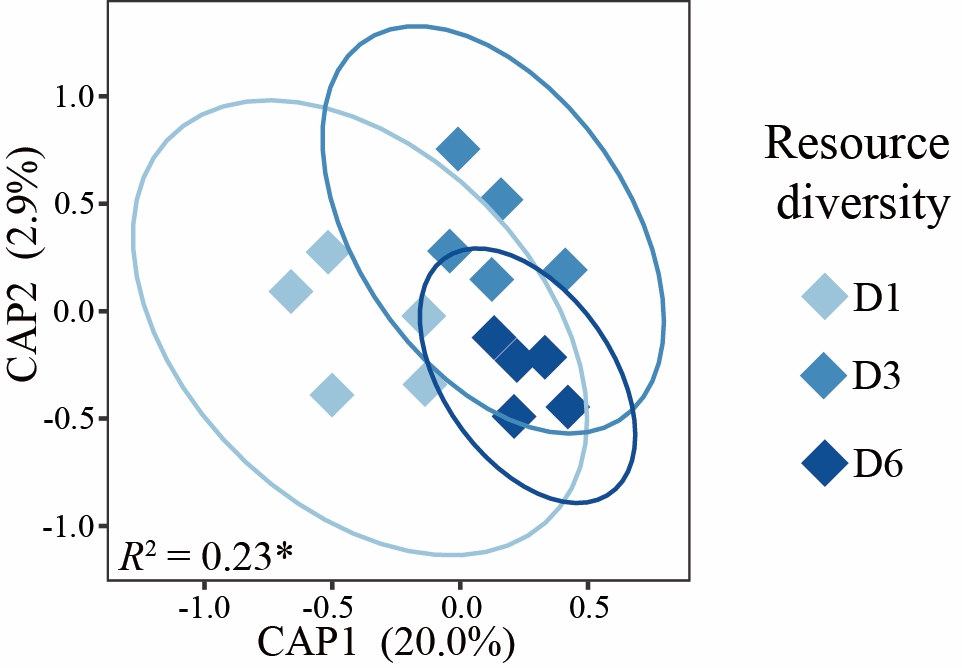


**Figure S24. Constrained analysis of the principal coordinates of the microbial communities in different resource diversity treatments.** The *R^2^* and *P*-value were determined by PERMANOVA and the symbol * denotes *P* < 0.05.

**Table S1. Initial physicochemical and microbial properties of the three soils used**

| Soil | pH | Ec  (μS cm^-1^) | TOC  (g kg^-1^) | TN  (g kg^-1^) | NH_4_^+^-N  (mg kg^-1^) | NO_3_^-^-N  (mg kg^-1^) | AP  (mg kg^-1^) | AK  (mg kg^-1^) | Abundance  (log_10_ copies g^-1^ soil) | Shannon | Richness | Evenness |
| --- | --- | --- | --- | --- | --- | --- | --- | --- | --- | --- | --- | --- |
| BS | 7.55±0.03a | 1991±97.90a | 63.1±1.79a | 7.65±0.17a | 11.6±0.22a | 965±16.65a | 490±43.54a | 908±27.79a | 10.6±0.07a | 5.86±0.07c | 1449±3.00c | 0.81±0.01c |
| FS | 7.56±0.02a | 335±300b | 11.2±0.61c | 1.41±0.01c | 8.33±0.14c | 169±8.14c | 427±14.21ab | 648±16.56b | 10.5±0.13a | 6.49±0.07a | 2011±37.23a | 0.85±0.01a |
| RS | 7.00±0.01b | 447±44.64b | 15.4±1.16b | 1.73±0.01b | 8.75±0.10b | 213±2.31b | 346±69.20b | 611±13.23b | 10.5±0.07a | 6.35±0.02b | 1859±18.82b | 0.84±0.00b |

Note: BS, FS, and RS represent black soil, fluvo-aquic soil, and red soil, respectively. Different letters represent significant differences at *P* < 0.05 according to one-way ANOVA and Duncan’s test. Data are presented as mean ± SD, n = 3.

**Table S2. Two-way ANOVA of the effects of plant diversity and soil type on soil ARG and MGE abundance.**

| Factor | df | Mean square | F | P |
| --- | --- | --- | --- | --- |
| Plant diversity | 2 | 4.10 | 442.02 | <0.001 |
| Soil type | 2 | 0.14 | 14.80 | <0.001 |
| Plant diversity * Soil type | 4 | 0.02 | 2.55 | 0.045 |

**Table S3. Primers used for high-throughput qPCR**

| No. | Gene | Drug Class | Type | Forward Primer | Reverse Primer |
| --- | --- | --- | --- | --- | --- |
| 1 | AAC(3)-Ia | Aminoglycoside | ARG | ACGTTCTGCCAAAGTTTGAG | ACTGCCGGATCGTCAC |
| 2 | AAC(3)-Ib | Aminoglycoside | ARG | CAGCGAGACGTTCATCGC | CACGCTTCAGGTGGCTAATC |
| 3 | AAC(3)-Id | Aminoglycoside | ARG | AGATAGTTATGCCCGCAACAAG | ACGCGCTGCGCCTATA |
| 4 | AAC(3)-Iic | Aminoglycoside | ARG | ACGGCATTCTCGATTGCTTT | CCGAGCTTCACGTAAGCATTT |
| 5 | Aac(3)-iid_iia_iie | Aminoglycoside | ARG | CGATGGTCGCGGTTGGTC | TCGGCGTAGTGCAATGCG |
| 6 | AAC(3)-IV | Aminoglycoside | ARG | CCAACACGACGCTGCATC | GCTGTCGCCACAATGTCG |
| 7 | AAC(3)-Via | Aminoglycoside | ARG | GTGTCCGTCGCCAAGGA | GGTGACGGCCTTGTCGA |
| 8 | AAC(3)-Xa | Aminoglycoside | ARG | GCAAGCGGTTCGTGACGTA | TCAGGTGCTCCTCGATCCAG |
| 9 | AAC(6')-Ib | Aminoglycoside | ARG | CGTCGCCGAGCAACTTG | CGGTACCTTGCCTCTCAAACC |
| 10 | aac(6')-ie-aph(2'')-ia | Aminoglycoside | ARG | CCAAGAGCAATAAGGGCATACCAA | GCCACACTATCATAACCACTACCG |
| 11 | AAC(6')-Ig | Aminoglycoside | ARG | GCGATGTTAGAAGCCTCAATTCG | CACACTTCGGCCTGTCGAA |
| 12 | AAC(6')-IIa | Aminoglycoside | ARG | CGACCCGACTCCGAACAA | GCACGAATCCTGCCTTCTCA |
| 13 | AAC(6')-IIc | Aminoglycoside | ARG | CAGTCTTTGGCTAATCCATCACAG | AACGAACCCGGCCTTCTC |
| 14 | AAC(6')-Ij | Aminoglycoside | ARG | ATGCCTGTATCTGAATCCCTGATG | GGCAATCGCTTGTTGAGTATCTG |
| 15 | AAC(6')-Im | Aminoglycoside | ARG | CGTGAGCATTATACAGAGCAATGG | CCATTTCCGTTCGTAGATATTGGC |
| 16 | AAC(6')-Ip | Aminoglycoside | ARG | GGGAATTATCGGAATAGCTCTTGG | TTGGGCTGTTCTTCCTAGCTAA |
| 17 | AAC(6')-Ir | Aminoglycoside | ARG | GCTATAACGATCAGCAGCAAGC | CGCGATGCATGGCATGAC |
| 18 | AAC(6')-Is | Aminoglycoside | ARG | AAGCTTACTCTGGCCTGATCATG | TGCCTGAACGTCGATATTCAGG |
| 19 | AAC(6')-Iv | Aminoglycoside | ARG | TTGGCTTATACCGACACCCA | CCCGTTGCGATACCTGAAC |
| 20 | AAC(6')-Iw | Aminoglycoside | ARG | TGCGTCAGTTACTTACACGAAC | CCTGATGCATTGCATGACTGA |
| 21 | AAC(6')-Iy | Aminoglycoside | ARG | GCCTCAATCCGCCACGATTA | ACGCGCTCTGTTTCCTCAAA |
| 22 | AAC(6')-Iz | Aminoglycoside | ARG | TGCGCCATGACTACGTGAAC | GACTGTCCGAAGCCAGTTCG |
| 23 | aacA_aphD | Aminoglycoside | ARG | AGAGCCTTGGGAAGATGAAGTTT | TTGATCCATACCATAGACTATCTCATC |
| 24 | aacA43 | Aminoglycoside | ARG | CTTGGCCTACATTAGATTCAGCTC | GCTCTCAATCTTTGATAGGAGCAG |
| 25 | aadA | Aminoglycoside | ARG | GTTGTGCACGACGACATCATT | GGCTCGAAGATACCTGCAAGAA |
| 26 | aadA10 | Aminoglycoside | ARG | ACAGGCACTCAACGTCATCG | CGCGGAGAACTCTGCTTTGA |
| 27 | aadA16 | Aminoglycoside | ARG | ACGGTGGCCTGAAGCC | GAATTGCAGTTCCCGTCTGG |
| 28 | aadA17 | Aminoglycoside | ARG | TGTACGGCTCCGCAGTG | CACGGAATGATGTCGTCGTG |
| 29 | aadA2 | Aminoglycoside | ARG | CAATGACATTCTTGCGGGTATC | GACCTACCAAGGCAACGCTATG |
| 30 | aadA21 | Aminoglycoside | ARG | ACGGCTCCGCAGTGGAT | GGCCACAGTAACCAACAAATCA |
| 31 | aadA5 | Aminoglycoside | ARG | ATCACGATCTTGCGATTTTGCT | CTGCGGATGGGCCTAGAAG |
| 32 | aadA6 | Aminoglycoside | ARG | CCATCGAGCGTCATCTGGAA | CCCGTCTGGCCGGATAAC |
| 33 | aadA7 | Aminoglycoside | ARG | CACTCCGCGCCTTGGA | TGTGGCGGGCTCGAAG |
| 34 | aadA9 | Aminoglycoside | ARG | CGCGGCAAGCCTATCTTG | CAAATCAGCGACCGCAGACT |
| 35 | ACC-1 | β-Lactamase | ARG | CACACAGCTGATGGCTTATCTAAAA | AATAAACGCGATGGGTTCCA |
| 36 | acrA | Multidrug | ARG | GGTCTATCACCCTACGCGCTATC | GCGCGCACGAACATACC |
| 37 | acrB | Multidrug | ARG | AGTCGGTGTTCGCCGTTAAC | CAAGGAAACGAACGCAATACC |
| 38 | AcrF | Multidrug | ARG | GCGGCCAGGCACAAAA | TACGCTCTTCCCACGGTTTC |
| 39 | acrR | Multidrug | ARG | GCGCTGGAGACACGACAAC | GCCTTGCTGCGAGAACAAA |
| 40 | ACTbeta-lac | β-Lactamase | ARG | AAGCCGCTCAAGCTGGA | GCCATATCCTGCACGTTGG |
| 41 | ADCbeta-lac | β-Lactamase | ARG | GGTATGGCTGTGGGTGTTATTCA | AGGCAAGGTTACCACTTGTATACG |
| 42 | adeA | Multidrug | ARG | CAGTTCGAGCGCCTATTTCTG | CGCCCTGACCGACCAAT |
| 43 | adeI | Multidrug | ARG | CAGTCTGGTTTGCAGTAACCA | CACTCCTACAACAACAGGCAA |
| 44 | AmpCbeta-lac | β-Lactamase | ARG | CAGGATCTGATGTGGGAGAACTA | TCGGGAACCATTTGTTGGC |
| 45 | ANT(2'')-Ia | Aminoglycoside | ARG | CCTGCTTGGTGGGCAGAC | CGGCACGCAAGACCTCAA |
| 46 | ANT(4') | Aminoglycoside | ARG | CCGACAACATTTCTACCATCCTT | ACCGAAGCGCTCGTCGTATA |
| 47 | ANT(4')-Ia | Aminoglycoside | ARG | GATGGCCGCTGACACATG | TCAACATTGCGCCATAGTGG |
| 48 | ANT(6) | Aminoglycoside | ARG | TACCTTATTGCCCTTGGAAGAGTTA | GGAACTATGTCCCTTTTAATTCTACAATCT |
| 49 | ANT(6)-Ia | Aminoglycoside | ARG | TCGCCATGAGCTGCTGA | CCTATCATACTCCGGATAGGCATA |
| 50 | ANT(6)-Ib | Aminoglycoside | ARG | AGAACATCCGACAGCACGTTC | CCAACCTTCCATGAAATCATTCGC |
| 51 | APH(2'')-Iva | Aminoglycoside | ARG | GACAGAACAATCAATCTCTATGGAATG | TGAGCAGTATCATAAGTTGAGTGAAAAG |
| 52 | APH(3')-Ia | Aminoglycoside | ARG | TGAACAAGTCTGGAAAGAAATGCA | CCTATTAATTTCCCCTCGTCAAAAA |
| 53 | APH(3'')-Ia | Aminoglycoside | ARG | TAACAGCGATCGCGTATTTCG | TCCGACTCGTCCAACATCAATA |
| 54 | APH(3')-Ib | Aminoglycoside | ARG | AACAGGTTTGGGAGGCGATG | CGCAACAAGCCTCTCCTGAA |
| 55 | APH(3')-Via | Aminoglycoside | ARG | TCTCATGGCGATATCACGGATAG | TTTCCTCCGATGCATCCTCTC |
| 56 | APH(3')-VIIa | Aminoglycoside | ARG | CTCTCTCATGGAGATATGAGCGCTA | AATCCGGTTCAAGTCCCAACATG |
| 57 | APH(3')-VIIIa | Aminoglycoside | ARG | TCGGTATCCCGGTTGTGAG | ACACGAGGTACGGGAATCC |
| 58 | APH(4)-Ia | Aminoglycoside | ARG | CGCTCCCGATTCCGGAA | CACAGTTTGCCAGTGATACACA |
| 59 | APH(4)-Ib | Aminoglycoside | ARG | GGGAACACCGTGCTCACC | GTTGGTCCCGTGCAGGTC |
| 60 | APH(6)-Ia | Aminoglycoside | ARG | CGCTGGGAGCTGAAGAGG | AGCATCGTGCTGCTCTCC |
| 61 | APH(6)-Ic | Aminoglycoside | ARG | CACGACAACGTGCTCGAC | CCGTCTTCGGCGAACCA |
| 62 | APH(6)-Id | Aminoglycoside | ARG | GCTCGGTCGTGAGAACAATCT | CAATTTCGGTCGCCTGGTAGT |
| 63 | APH(9)-Ib | Aminoglycoside | ARG | GCTATGTGCTGGTGGACTGG | GGAACCACTCGACGAACTCG |
| 64 | APH3-III | Aminoglycoside | ARG | CAGAAGGCAATGTCATACCACTTG | GACAGCCGCTTAGCCGAA |
| 65 | APHA3 | Aminoglycoside | ARG | AAAAGCCCGAAGAGGAACTTG | CATCTTTCACAAAGATGTTGCTGTCT |
| 66 | apmA | Aminoglycoside | ARG | GGCGCACATGCATTCATCA | CTATACTCCAGTCCCACCATTTGA |
| 67 | armA | Aminoglycoside | ARG | TCTTCGACGAATGAAAGAGTCG | GCTAATGGATTGAAGCCACAACC |
| 68 | arr-2 | Rifamycin | ARG | TTGGCGATTGGTGACTTGCTAA | ATCGTCTTCGAACGGTCCTG |
| 69 | arr-3 | Rifamycin | ARG | GATCGTCTTCGAACGGTCCTG | TTTGGCGATTGGTGACTTGCT |
| 70 | acA | Peptide | ARG | ATCCGCGGCACCCTGA | CCTGCTTGATGGACTTGATGAAGA |
| 71 | BELbeta-lac | β-Lactamase | ARG | ATGTCCATGGCACAGACTGTG | CCTGTCTTGTCACCCGTTACC |
| 72 | bl1acc | β-Lactamase | ARG | TGTTATCCGTGATTACCTGTCTGG | CTCAGCGAGCCAACTTCAAATA |
| 73 | Bla1 | β-Lactamase | ARG | GCAAGTTGAAGCGAAAGAAAAGA | TACCAGTATCAATCGCATATACACCTAA |
| 74 | BlaBbeta-lac | β-Lactamase | ARG | CGTGCCGGAGGTCTTGAATA | GGGATAGTAAACCTGAAACTCGGA |
| 75 | blaSFO | β-Lactamase | ARG | CCGCCGCCATCCAGTA | GGGCCGCCAAGATGCT |
| 76 | blaZbeta-lac | β-Lactamase | ARG | TGCTTAATTTTCCATTTGCGATAAG | GGAGATAAAGTAACAAATCCAGTTAGATATGA |
| 77 | CARBbeta-lac | β-Lactamase | ARG | TGATTTGAGGGATACGACAACTCC | CTGTAATACTCCGAGCACCAA |
| 78 | CARB-2 | β-Lactamase | ARG | TTGTGACCTATTCCCCTGTAATAGAA | TGCGAAGCACGCATCATC |
| 79 | cat | Phenicol | ARG | ATCGGCCAGACTGGATATCGA | CACAGCTCCAGTTGCAACAAC |
| 80 | cat(pC221) | Phenicol | ARG | AATGACCGTATGCTGCAAGAAG | TTTGCCTGCTATGGCATTCTG |
| 81 | catB2 | Phenicol | ARG | GCTACTATTCCGGCTATTACCATG | GGGCTCCTCGTTCATGTAGA |
| 82 | catB3 | Phenicol | ARG | GCACTCGATGCCTTCCAAAA | AGAGCCGATCCAAACGTCAT |
| 83 | catB8 | Phenicol | ARG | CACTCGACGCCTTCCAAAG | CCGAGCCTATCCAGACATCATT |
| 84 | catB9 | Phenicol | ARG | CACCTTATGAAGTGGTCGGTTCA | GTCTGATGAACACAGAGACTGCA |
| 85 | catI | Phenicol | ARG | GGGTGAGTTTCACCAGTTTTGATT | CACCTTGTCGCCTTGCGTATA |
| 86 | catII | Phenicol | ARG | CCTGGAACCGCAGAGAACA | CGGAACTCCGGAAACTGATTAAC |
| 87 | catIII | Phenicol | ARG | CTGATTGCTCAGGCCGTGAA | ATGAGTATGGGCAACTCAGTGC |
| 88 | catP | Phenicol | ARG | CCTTTGGACTGAGTGTAAGTCTGA | TAAAGCCATCGAAGGTTGACCA |
| 89 | catQ | Phenicol | ARG | AGGTGCACTTACAGTATGACTGC | AACGTGGGAAGTTCTCGTCATAC |
| 90 | CcrA | β-Lactamase | ARG | GCAGCGTTGCTGGACACA | GTTCGGGATAAACGTGGTGACT |
| 91 | CcrAbeta-lac | β-Lactamase | ARG | CACTGGCACGGCGATTGTA | CGGCAGCCAAACCACGATA |
| 92 | cefa_qacelta | Multidrug | ARG | TAGTTGGCGAAGTAATCGCAAC | TGCGATGCCATAACCGATTATG |
| 93 | ceoA | Multidrug | ARG | ATCAACACGGACCAGGACAAG | GGAAAGTCCGCTCACGATGA |
| 94 | cepAbeta-lac | β-Lactamase | ARG | AGTTGCGCAGAACAGTCCTCTT | TCGTATCTTGCCCGTCGATAAT |
| 95 | CfrGroup | Multidrug | ARG | GCAAAATTCAGAGCAAGTTACGAA | AAAATGACTCCCAACCTGCTTTAT |
| 96 | CfxAbeta-lac | β-Lactamase | ARG | TCATTCCTCGTTCAAGTTTTCAGA | TGCAGCACCAAGAGGAGATGT |
| 97 | classCbeta-lac | β-Lactamase | ARG | CTGGCGCATACCTGGATTAC | GCCAGTTCAGCATCTCCCA |
| 98 | cmlA1 | Phenicol | ARG | TAGGAAGCATCGGAACGTTGAT | CAGACCGAGCACGACTGTTG |
| 99 | cmlA5 | Phenicol | ARG | GCGCTCTTCGAGGATTCG | CCGCCCAAGCAGAAGTAGAC |
| 100 | cmlv | Phenicol | ARG | GCCCTCATCACCGTCTTCG | GGACGTTGGCGATGGAGAG |
| 101 | cmr | Multidrug | ARG | CGGCATCGTCAGTGGAATT | CGGTTCCGAAAAAGATGGAA |
| 102 | cmx | Phenicol | ARG | GCGATCGCCATCCTCTGT | TCGACACGGAGCCTTGGT |
| 103 | CMYbeta-lac | β-Lactamase | ARG | AAAGCCTCATGGGTGCATAAA | ATAGCTTTTGTTTGCCAGCATCA |
| 104 | CMY_moxbeta-lac | β-Lactamase | ARG | CTATGTCAATGTGCCGAAGCA | GGCTTGTCCTCTTTCGAATAGC |
| 105 | CphAbeta-lac | β-Lactamase | ARG | GCGAGCTGCACAAGCTGAT | CGGCCCAGTCGCTCTTC |
| 106 | cphA2 | β-Lactamase | ARG | GTAACGCCTACTGGAAGTCCA | CAGCTTCTCCTTGAGAATGCAG |
| 107 | cro | MGE | MGE | AGATGTTATCGACCACTTCGGA | CCGCTTGGCGATAAGCG |
| 108 | CTX-Mbeta-lac | β-Lactamase | ARG | GCGATAACGTGGCGATGAAT | GTCGAGACGGAACGTTTCGT |
| 109 | CTX-M-1_3_15 | β-Lactamase | ARG | CGTACCGAGCCGACGTTAA | CAACCCAGGAAGCAGGCA |
| 110 | dfrA1 | Diaminopyrimidine | ARG | GGAATGGCCCTGATATTCCA | AGTCTTGCGTCCAACCAACAG |
| 111 | dfrA10 | Diaminopyrimidine | ARG | CTTCAACTATCACAGAGCACGAAG | TCTACCGGTACATACACATCAGC |
| 112 | dfrA12 | Diaminopyrimidine | ARG | CCTCTACCGAACCGTCACACA | GCGACAGCGTTGAAACAACTAC |
| 113 | dfrA14 | Diaminopyrimidine | ARG | CGGATCATGTCATTGTTTCAGG | ATGTTAGAGGCGAAGTCTTGG |
| 114 | dfrA15 | Diaminopyrimidine | ARG | AGGCCGAAAGACTTTCGAGTC | TCACCTTCTGGCTCAATGTCG |
| 115 | dfrA17 | Diaminopyrimidine | ARG | CGGGAACGGCCCTGATATTCC | CGTGTTGCGACCGCATACTTTC |
| 116 | dfrA18 | Diaminopyrimidine | ARG | GGAGCGAATCAAGGAGAAAGGAA | GCAATGCGTTGATCGGTATTCTC |
| 117 | dfrA21 | Diaminopyrimidine | ARG | TTGTTTCAACGCTGTCGCA | GGTTTCGGTTGAGACAAGCTC |
| 118 | dfrA22 | Diaminopyrimidine | ARG | CAGCCGAACACGGCAAAG | CGGAGTGCGTGTACGTGA |
| 119 | dfrA25 | Diaminopyrimidine | ARG | TCAAACTGGACAGCGGCTA | GTCGATTGTCGACACATGCA |
| 120 | dfrA27 | Diaminopyrimidine | ARG | GCCGCTCAGGATCGGTA | GTCGAGATATGTAGCGTGTCG |
| 121 | dfrA5 | Diaminopyrimidine | ARG | CCATGGAGTGCCAAAGGTG | CACCTTTGGCACTCCATGG |
| 122 | dfrA7 | Diaminopyrimidine | ARG | GTAATCGGTAGTGGTCCTGA | ATCAGGACCACTACCGATTAC |
| 123 | dfrA8 | Diaminopyrimidine | ARG | GGTCGCACCTGCATCGTTA | AGCGCCACCAATGACGTAG |
| 124 | dfrB4 | Diaminopyrimidine | ARG | CGGTTCGCATTCCCATCAAA | CGCAGTCATGGGATAAATCTGG |
| 125 | dfrBmulti | Diaminopyrimidine | ARG | ACCAAGGCAGAAGTGAAGTCA | GGTGAGCCTCAGACTCGAC |
| 126 | dfrC | Diaminopyrimidine | ARG | GTCGCTCACGATAAACAAAGAGTC | CCCTTCATGGTGAAATGAAGCTTG |
| 127 | dfrG | Diaminopyrimidine | ARG | TCAATCGGAAGAGCCTTACCTGA | TGGGCAAATACCTCATTCCATTCC |
| 128 | dfrK | Diaminopyrimidine | ARG | TGCTGCGATGGATAAGAACAG | CTTCCAGGTAATGCTCTTCCG |
| 129 | DHAbeta-lac | β-Lactamase | ARG | TGGCCGCAGCAGAAAGA | CCGTTTTATGCACCCAGGAA |
| 130 | EAE_05855 | MGE | MGE | CCCATCACCGCTGAACTGG | TGGGCGCTGCCATCTAAAC |
| 131 | emrB_qacA | Multidrug | ARG | AGAACGTAGCGACTGATAAAATGCT | CTTTTCTCTAACCGTACATTATCTACGATAAA |
| 132 | emrD | Multidrug | ARG | CTCAGCAGTATGGTGGTAAGCATT | ACCAGGCGCCGAAGAAC |
| 133 | EreA | MLSB | ARG | GATAATTCTGCTGGCGCACA | GCAGGCGTGGTCACAAC |
| 134 | EreB | MLSB | ARG | TCGTATATGGCGGGCGTAGTA | GGTCCAAGATGGGTGAATGCA |
| 135 | Erm(34) | MLSB | ARG | AAAGCGGTTTACAAGCGTTTCG | GGGTGCTCTAGGGTTGTTTAGTG |
| 136 | Erm(35) | MLSB | ARG | CCTTCAGTCAGAACCGGCAA | GCTGATTTGACAGTTGGTGGTG |
| 137 | Erm(36) | MLSB | ARG | GGCGGACCGACTTGCAT | TCTGCGTTGACGACGGTTAC |
| 138 | Erm(42) | MLSB | ARG | TGTTGAGATTGGGCCTGGA | CTAAGGGTGGGTTCTCACTATCTA |
| 139 | Erm(K) | MLSB | ARG | GTTTGATATTGGCATTGTCAGAGAA | ACCATTGCCGAGTCCACTTT |
| 140 | erm(O) | MLSB | ARG | TGATGACGGCTCAGTGG | GTGCACCAGCGCCTGA |
| 141 | ErmA | MLSB | ARG | TCGTTGAGAAGGGATTTGCGA | TTGCATGCTTCAAAGCCTGTC |
| 142 | ermA_ermTR | MLSB | ARG | ACATTTTACCAAGGAACTTGTGGAA | GTGGCATGACATAAACCTTCATCA |
| 143 | ErmB | MLSB | ARG | GAACACTAGGGTTGTTCTTGCA | CTGGAACATCTGTGGTATGGC |
| 144 | ErmD | MLSB | ARG | TTTCCGGACAGCATTTGATGC | TCCACTGCCAATACCTTACCG |
| 145 | ErmE | MLSB | ARG | GTCACGCAGCTGGAGTTCG | CGGTGAAGCACAGCTCGAC |
| 146 | ErmF | MLSB | ARG | CAGCTTTGGTTGAACATTTACGAA | AAATTCCTAAAATCACAACCGACAA |
| 147 | ErmG | MLSB | ARG | CCCTTGAATTAGTACAGAGGTG | GCAAACTCGTATTCCACGA |
| 148 | ErmH | MLSB | ARG | GGAGTGAGGCTGACCGTAGAAG | ATCGGCGAAACGCACAAA |
| 149 | ErmQ | MLSB | ARG | TGAAAGCCATGCGTCTGAC | TTCAGCTGGCAGCTTAAGC |
| 150 | ErmS | MLSB | ARG | GAGTACGCCCGCAAACG | GCGTTCGATCCGGAGGA |
| 151 | ErmT | MLSB | ARG | GAAGGGTGTCTTTTTAATACAATTAACGA | GTTCACTAGCACTATTTTTAATGACAGAAGT |
| 152 | ErmX | MLSB | ARG | GCTCAGTGGTCCCCATGGT | ATCCCCCCGTCAACGTTT |
| 153 | ErmY | MLSB | ARG | TTGTCTTTGAAAGTGAAGCAACAGT | TAACGCTAGAGAACGATTTGTATTGA |
| 154 | fabK | Other | ARG | CAGGAGCAGGAAATCCAAGC | CCAGCTTCCATTCCTTCTGC |
| 155 | exA | Phenicol | ARG | TGGTGTGGCTGTTGCAATCTTA | CCAAGGTACAAAGCACCTTGGA |
| 156 | floR | Phenicol | ARG | AACCCGCCCTCTGGATCA | GCCGTCGAGAAGAAGACGAA |
| 157 | folA | Other | ARG | CGAGCAGTTCCTGCCAAAG | CCCAGTCATCCGGTTCATAATC |
| 158 | FosB | Fosfomycin | ARG | CTTGCAGGCCTATGGATTGC | TCTGTTCTCAAGTGTGCCAGTA |
| 159 | FosX | Fosfomycin | ARG | AGCTGGTTTGTGGATTTGCA | CCACACCGAGAGCTTTAATCCG |
| 160 | FOXbeta-lac | β-Lactamase | ARG | CCTACGGCTATTCGAAGGAAGATAA | CCGGATTGGCCTGGAAGC |
| 161 | GESbeta-lac | β-Lactamase | ARG | GCAATGTGCTCAACGTTCAAG | GTGCCTGAGTCAATTCTTTCAAAG |
| 162 | GOBbeta-lac | β-Lactamase | ARG | CTTGGGCTTGAATGCTCAGGTA | TGTATGGTCGTAGTGAGCCTGA |
| 163 | HERAbeta-lac | β-Lactamase | ARG | GGGCAACCGCATTCTGAC | GCATCTCCCACTTTATCGTCAC |
| 164 | IMIbeta-lac | β-Lactamase | ARG | ACATCTACACCTGCAGCAGTAG | AATCGCTTGGTACGCTAGCA |
| 165 | IMIRbeta-lac | β-Lactamase | ARG | AGCCGGACTAGAGCTTCATG | GGCAGAACTCATCATCTGCAAA |
| 166 | IMPbeta-lac | β-Lactamase | ARG | GGAATAGAGTGGCTTAATTC | GGTTTAACAAAACAACCACC |
| 167 | IncHI2-smr0018 | MGE | MGE | ATAATGATTCACCGGGGTAG | CTTCAGGCTATCGTTTCG |
| 168 | IncI1_repI1 | MGE | MGE | CGAAAGCCGGACGGCAGAA | TCGTCGTTCCGCCAAGTTCGT |
| 169 | IncN_korA | MGE | MGE | GGAACGTTTGTAYCTTGTATTG | ACTCACTATCTTCTGTTGATTG |
| 170 | IncN_oriT | Plasmid-inc | MGE | TTGGGCTTCATAGTACCC | GTGTGATAGCGTGATTTATGC |
| 171 | IncN_rep | Plasmid-inc | MGE | AGTTCACCACCTACTCGCTCCG | CAAGTTCTTCTGTTGGGATTCCG |
| 172 | IncP_oriT | Plasmid-inc | MGE | CAGCCTCGCAGAGCAGGAT | CAGCCGGGCAGGATAGGTGAAGT |
| 173 | IncQ_oriT | Plasmid-inc | MGE | TTCGCGCTCGTTGTTCTTCGAGC | GCCGTTAGGCCAGTTTCTCG |
| 174 | IncW_trwAB | Plasmid-inc | MGE | AGCGTATGAAGCCCGTGAAGGG | AAAGATAAGCGGCAGGACAATAACG |
| 175 | INDbeta-lac | β-Lactamase | ARG | CGCCTGTTAAACCCAACCTGTA | CGCTCTGTCATCATGAGAGTGG |
| 176 | intI1_337old | Integrase | MGE | GCCTTGATGTTACCCGAGAG | GATCGGTCGAATGCGTGT |
| 177 | intl2 | Integrase | MGE | TGCTTTTCCCACCCTTACC | GACGGCTACCCTCTGTTATCTC |
| 178 | intl3_339old | Integrase | MGE | GCCACCACTTGTTTGAGGA | GGATGTCTGTGCCTGCTTG |
| 179 | IS1111 | Insertional | MGE | GTCTTAAGGTGGGCTGCGTG | CCCCGAATCTCATTGATCAGC |
| 180 | IS1133 | Insertional | MGE | GCAGCGTCGGGTTGGA | ACGCGTTCGAACAACTGTAATG |
| 181 | IS1247 | MGE | MGE | CGGCCGTCACTGACCAA | TCGGCAGGTTGGTGACG |
| 182 | IS15DI | MGE | MGE | CAATACCTTTGATGGTGGCGTAAG | CTTACGCCACCATCAAAGGTATTG |
| 183 | IS200-1 | MGE | MGE | CCAAATACCGAAGACAAGCGTTC | CCAAACTGCTCGTAAAGCATCAG |
| 184 | IS200-2 | MGE | MGE | GCACACCCGATGGAACTGTAAA | TCGGCGGGATCTCCAGAAG |
| 185 | IS21-ISAs29 | MGE | MGE | GGTCCGTCAGGCACAAGTC | GGGATCGTATCGGCAAGCC |
| 186 | IS256 | MGE | MGE | CTTGCGCATCATTGGATGATGG | AAGAACGGCTCCAATTAAGCGA |
| 187 | IS26 | MGE | MGE | ATGGATGAAACCTACGTGAAGGTC | CGGTACTTAATCTGTCGGTGTTCA |
| 188 | IS3 | MGE | MGE | CGGTCTGAGCTTCGGGAA | AGAACTGTCACTCCGGTCTG |
| 189 | IS5/IS1182 | MGE | MGE | TTCTCGAAGAATCGCCATGGC | GCTTTGGATCGCTCCAATCGA |
| 190 | IS6/257 | MGE | MGE | ATATCGTGCCATTGATGCAGAG | ACCATTGCTACCTTCGTTGAAG |
| 191 | IS6100 | MGE | MGE | CGCACCGGCTTGATCAGTA | CTGCCACGCTCAATACCGA |
| 192 | IS613 | Transposase | MGE | AGGTTCGGACTCAATGCAACA | TTCAGCACATACCGCCTTGAT |
| 193 | IS630 | MGE | MGE | CCGCCACCAGTGTGATGG | TTGGCGCTGACTGGATGC |
| 194 | IS91 | MGE | MGE | GGATGCCACTGCTGGTCA | ACAGTGGATACAGTATCTGCTGAG |
| 195 | ISAba3-Acineto | Insertional | MGE | TCAGAGGCAGCGGTATACGA | GGTTGATTCAGTTAAAGTACGTAAAACTTT |
| 196 | ISCR1 | MGE | MGE | ATGGTTTCATGCGGGTT | CTGAGGGTGTGAGCGAG |
| 197 | ISEcp1 | MGE | MGE | CATGCTCTGCGGTCACTTC | GACGCACCTTCTTGATGACC |
| 198 | ISEfm1-Entero | Insertional | MGE | AGGTGTCCATGACGTGAAAGTG | TCCTTTGTCCCCTAGGATATTGG |
| 199 | ISPps1-pseud | Insertional | MGE | CACACTGCAAAAACGCATCCT | TGTCTTTGGCGTCACAGTTCTC |
| 200 | ISSm2-Xanthob | Insertional | MGE | TGGATCGACCGGTTCCAT | GCTGACCGAGCTGTCCATGT |
| 201 | KPCbeta-lac | β-Lactamase | ARG | GCCGCCAATTTGTTGCTGAA | GCCGGTCGTGTTTCCCTTT |
| 202 | L1beta-lac | β-Lactamase | ARG | CACCGGGTTACCAGCTGAAG | GCGAAGCTGCGCTTGTAGTC |
| 203 | LENbeta-lac | β-Lactamase | ARG | TGTTCGCCTGTGTGTTATCTCC | GCAGCACTTTAAAGGTGCTCAC |
| 204 | mrA | MLSB | ARG | TTCAGATGCAATGGCGTTTG | ATAATCGGGAACATAATGAGCATAACTAC |
| 205 | lncF_FIC | MGE | MGE | GTGAACTGGCAGATGAGGAAGG | TTCTCCTCGTCGCCAAACTAGAT |
| 206 | nuA | MLSB | ARG | TGACGCTCAACACACTCAAAAA | TTCATGCTTAAGTTCCATACGTGAA |
| 207 | nuB | MLSB | ARG | GGATCGTTTACCAAAGGAGAAGG | AGCATAGCCTTCGTATCAGGAA |
| 208 | nuC | MLSB | ARG | GGGTGTAGATGCTCTTCTTGGA | CTTTACCCGAAAGAGTTTCTACCG |
| 209 | nuF | MLSB | ARG | ATACCGGTCATTTCCACTTGGC | GCATCAGGCTGATGAGGTTCAA |
| 210 | saC | Multidrug | ARG | AAACGGCGTGAAAGTATCAGG | TTGTGGTGATGTAACGGATGC |
| 211 | marR | Multidrug | ARG | GCTGTTGATGACATTGCTCACA | CGGCGTACTGGTGAAGCTAAC |
| 212 | MCR-1.1 | Peptide | ARG | CACATCGACGGCGTATTCTG | CAACGAGCATACCGACATCG |
| 213 | MCR-2.1 | Peptide | ARG | CGGCGTACTTTAAGCGTTATGATG | GCATTTGGCATACCATGCAGATAG |
| 214 | mdtA | Multidrug | ARG | ACAAGCCCAGGGCCAAC | CCTTAATGGTGCCTTCGGTTTC |
| 215 | mdtE | Multidrug | ARG | CGTCGGCGCACTCGTT | TCCAGACGTTGTACGGTAACCA |
| 216 | mdtG | Multidrug | ARG | TTCCAGCCGGTCAGCAA | GACATCTCCCGCGAGTTCG |
| 217 | mdtH | Fluoroquinolone | ARG | ATGCTGGCTGTACAAGTGATG | CACTCCAGCGGGCGATA |
| 218 | MdtK | Multidrug | ARG | TCGGGCATCCCGTTTATGATC | GTAGGCTGCGCATAATACCCA |
| 219 | mecA | β-Lactamase | ARG | GGTTACGGACAAGGTGAAATACTGAT | TGTCTTTTAATAAGTGAGGTGCGTTAATA |
| 220 | mef(B) | MLSB | ARG | CCGATAGGCTTACTTGTTGCAG | AGTCCACTTGCGGTTTCATTG |
| 221 | mel_1 | Multidrug | ARG | TAATTATCGCAGCAGCTGGTTC | GTTCCCAAACGGAGTATAAGAGTG |
| 222 | mel_2 | Multidrug | ARG | GGCAAGCTAGGTGTTGAGC | ATTGCTCAACACCTAGCTTGC |
| 223 | mepA | Multidrug | ARG | ATCGGTCGCTCTTCGTTCAC | ATAAATAGGATCGAGCTGCTGGAT |
| 224 | MexA | Multidrug | ARG | AGGACAACGCTATGCAACGAA | CCGGAAAGGGCCGAAAT |
| 225 | MexB | Multidrug | ARG | CTGGAGATCGACGACGAGAAG | GAAATCGTTGACGTAGCTGGAA |
| 226 | MexE | Multidrug | ARG | GGTCAGCACCGACAAGGTCTAC | AGCTCGACGTACTTGAGGAACAC |
| 227 | MIRbeta-lac | β-Lactamase | ARG | CGGTCTGCCGTTACAGGTG | AAAGACCCGCGTCGTCATG |
| 228 | mobA | MGE | MGE | GCTTCCCGTAACGAGGTAGT | CCTTGAACGGTATCAGCACG |
| 229 | mphA | MLSB | ARG | TCAGCGGGATGATCGACTG | GAGGGCGTAGAGGGCGTA |
| 230 | mphB | MLSB | ARG | CGCAGCGCTTGATCTTGTAG | TTACTGCATCCATACGCTGCTT |
| 231 | msrA | Multidrug | ARG | CTGCTAACACAAGTACGATTCCAAAT | TCAAGTAAAGTTGTCTTACCTACACCATT |
| 232 | msrC | Multidrug | ARG | TCAGACCGGATCGGTTGTC | CCTATTTTTTGGAGTCTTCTCTCTAATGTT |
| 233 | msrE | Multidrug | ARG | CGGCAGATGGTCTGAGCTTAAA | CGCACTCTTCCTGCATAAAGGA |
| 234 | mtrD | Multidrug | ARG | CGGAGTCCATCGACCATTTG | ATCGTCGGCAAGGAGAATCA |
| 235 | mtrE | Multidrug | ARG | CGATGTGTCGTTTTGGAAGGT | CCTGCACCATGATTCCTCAATA |
| 236 | multidrug | Multidrug | ARG | AATTTTGCCGATTATTGCTGAAA | GATTGTCATCATTCGTTTATCACCAA |
| 237 | NDMbeta-lac | β-Lactamase | ARG | GGCCACACCAGTGACAATATCA | CAGGCAGCCACCAAAAGC |
| 238 | nimE | Multidrug | ARG | TGCGCCAAGATAGGGCATA | GTCGTGAATTCGGCAGGTTTA |
| 239 | nisB | Other | ARG | GGGAGAGTTGCCGATGTTGTA | AGCCACTCGTTAAAGGGCAAT |
| 240 | norA | Multidrug | ARG | ATCGCCGTTTGGTGGTACG | TCCACCAATCCCTGGTCCTAAA |
| 241 | OCHbeta-lac | β-Lactamase | ARG | GGCGACTTGCGCCGTAT | TTTTCTGCTCGGCCATGAG |
| 242 | oleC | MLSB | ARG | CCCGGAGTCGATGTTCGA | GCCGAAGACGTACACGAACAG |
| 243 | OprD | Multidrug | ARG | ATGAAGTGGAGCGCCATTG | GGCCACGGCGAACTGA |
| 244 | optrA | Multidrug | ARG | GGTGGATGAAGTCCGTACGG | AGGTTAGACCTCCAAGAGCCA |
| 245 | oqxA | Multidrug | ARG | GAGTCAACCTACCTCCACTATCA | GCTGCGAGTTATCCAGCAG |
| 246 | orf37-IS26 | Insertional | MGE | GCCGGGTTGTGCAAATAGAC | TGGCAATCTGTCGCTGCTG |
| 247 | orf39-IS26 | Insertional | MGE | GCGCGTCGAGCATCAATAG | CAGTTGTGCTGCTGGTGGTC |
| 248 | OXA-10 | β-Lactamase | ARG | CGACCGAGTATGTACCTGCTTC | TCAAGTCCAATACGACGAGCTA |
| 249 | OXY-1-1 | β-Lactamase | ARG | AAAGGTGACCGCATTCGC | CCAGCGTCAGCTTGCG |
| 250 | OXY-2-1 | β-Lactamase | ARG | CGTTCAGGCGGCAGGTT | GCCGCGATATAAGATTTGAGAATT |
| 251 | pAKD1-IncP-1β | Plasmid-rep | MGE | GGTAAGATTACCGATAAACT | GTTCGTGAAGAAGATGTA |
| 252 | Pambl-1-F_377old | Plasmid-rep | MGE | CAGGCTCTTAATGTGATA | TTATGCTCAATACTCGTG |
| 253 | pbp | β-Lactamase | ARG | CCGGTGCCATTGGTTTAGA | AAAATAGCCGCCCCAAGATT |
| 254 | Pbp5 | β-Lactamase | ARG | GGCGAACTTCTAATTAATCCTATCCA | CGCCGATGACATTCTTCTTATCTT |
| 255 | pBS228-IncP-1α | Plasmid-rep | MGE | CAATCCATCGACAATCAC | GACAATCAGCTACTTCAC |
| 256 | PDCbeta-lac | β-Lactamase | ARG | CGCCGTACAACCGGTGAT | GAAGTAATGCGGTTCTCCTTTCA |
| 257 | penA | β-Lactamase | ARG | GCGTGTAGCCGGCAATG | AGACGGTAACGTATAACTTTTTGAAAGA |
| 258 | PER-1 | β-Lactamase | ARG | GCAAATGAAGCGCAGATGC | GACCACAGTACCAGCTGGTA |
| 259 | pica | MLSB | ARG | GCAATCGAGGCGGTGTTC | TTGCCGCAGCCAATTCA |
| 260 | pikR2 | MLSB | ARG | TCGTGGGCCAGGTGAAGA | TTCCCCTTGCCGGTGAA |
| 261 | pmrA | Fluoroquinolone | ARG | TTTGCAGGTTTTGTTCCTAATGC | GCAGAGCCTGATTTCTCCTTTG |
| 262 | qacA_B | Fluoroquinolone | ARG | AAGGGCCACTGCATTAGCTG | CCAGTCCAATCATGCCTGCA |
| 263 | qacF_H | Multidrug | ARG | TCGCAACATCCGCATTAAAA | ATGGATTTCAGAACCAGAGAAAGAAA |
| 264 | qacH_351 | Multidrug | ARG | GTCGGTGTTGCTTATGCAGTCT | CAACCAGGCAATGGCTGTAA |
| 265 | QepA_1_2 | Fluoroquinolone | ARG | GGGCATCGCGCTGTTC | GCGCATCGGTGAAGCC |
| 266 | QnrA | Fluoroquinolone | ARG | AGGATTTCTCACGCCAGGATT | CCGCTTTCAATGAAACTGCAA |
| 267 | QnrB4 | Fluoroquinolone | ARG | TCACCACCCGCACCTG | GGATATCTAAATCGCCCAGTTCC |
| 268 | QnrB46_47_48 | Fluoroquinolone | ARG | CGACGTTCAGTGGTTCAGATCTC | GCCAAGCCGCTCCATGAG |
| 269 | QnrB-bob_resign | Fluoroquinolone | ARG | GCGACGTTCAGTGGTTCAGA | GCTGCTCGCCAGTCGAA |
| 270 | QnrD | Fluoroquinolone | ARG | CGCTGGAATGGCACTGTGA | GCTCTCCATCCAACTTCACTCC |
| 271 | QnrS1_S3_S5 | Fluoroquinolone | ARG | CCACTTTGATGTCGCAGATCTTC | CCCTCTCCATATTGGCATAGGAAA |
| 272 | QnrS2 | Fluoroquinolone | ARG | TCCCGAGCAAACTTTGCCAA | GGTGAGTCCCTATCCAGCGA |
| 273 | Qnrvc1_vc3_vc6 | Fluoroquinolone | ARG | CTCACATCAGGACTTGCAAGAA | ATGAAGCATCTCGAAGATCAGC |
| 274 | Qnrvc4_vc5_vc7 | Fluoroquinolone | ARG | TTCCTTTAAACGGGCAAACCTC | CGATACCTGATTCATGAAGCTAGC |
| 275 | ROB-1 | β-Lactamase | ARG | GCAAAGGCATGACGATTGC | CGCGCTGTTGTCGCTAAA |
| 276 | SAT-4 | Nucleoside | ARG | GAATGGGCAAAGCATAAAAACTTG | CCGATTTTGAAACCACAATTATGATA |
| 277 | SHV-11 | β-Lactamase | ARG | TTGACCGCTGGGAAACGG | TCCGGTCTTATCGGCGATAAAC |
| 278 | SMEbeta-lac | β-Lactamase | ARG | GAGGAAGACTTTGATGGGAGGATTG | CGCTATATTGCAATGCAGCAGAAG |
| 279 | spec_aph | Aminoglycoside | ARG | GGTGCTGATATGAATGCCTTTGG | CATTGGGCGCATCAATAAATGG |
| 280 | str | Aminoglycoside | ARG | AATGAGTTTTGGAGTGTCTCAACGTA | AATCAAAACCCCTATTAAAGCCAAT |
| 281 | strA | Aminoglycoside | ARG | CCGGTGGCATTTGAGAAAAA | GTGGCTCAACCTGCGAAAAG |
| 282 | sugE | Multidrug | ARG | CTTAGTTATTGCTGGTCTGCTGGA | GCATCGGGTTAGCGGACTC |
| 283 | sul1 | Sulfonamide | ARG | GCCGATGAGATCAGACGTATTG | CGCATAGCGCTGGGTTTC |
| 284 | sul2 | Sulfonamide | ARG | TCATCTGCCAAACTCGTCGTTA | GTCAAAGAACGCCGCAATGT |
| 285 | sul3 | Sulfonamide | ARG | CGCGCTCAAGGCAGATG | GGGAATGCCATCTGCCTTG |
| 286 | sulA_folP | Sulfonamide | ARG | CAGGCTCGTAAATTGATAGCAGAAG | CTTTCCTTGCGAATCGCTTT |
| 287 | TEMbeta-lac | β-Lactamase | ARG | CGCCGCATACACTATTCTCAG | GCTTCATTCAGCTCCGGTTC |
| 288 | tet(38) | Tetracycline | ARG | AAGCGACATTAGCCGGTTTAG | CTGCTCGTACTTAAGCCAAGG |
| 289 | tet(39) | Tetracycline | ARG | TATAGCGGGTCCGGTAATAGGTG | CCATAACGATCCTGCCCATAGATAAC |
| 290 | tet(40) | Tetracycline | ARG | CTGTCCGTGCGCAATATATCC | GGATATATTGCGCACGGACAG |
| 291 | tet(44) | Tetracycline | ARG | CTCATGTAGATGCAGGAAAGACG | GTAACTGCTGCCTGAATTGTGA |
| 292 | tet32 | Tetracycline | ARG | CCATTACTTCGGACAACGGTAGA | CAATCTCTGTGAGGGCATTTAACA |
| 293 | tet36 | Tetracycline | ARG | AGAATACTCAGCAGAGGTCAGTTCC | TGGTAGGTCGATAACCCGAAAAT |
| 294 | tetA | Tetracycline | ARG | CTCACCAGCCTGACCTCGAT | CACGTTGTTATAGAAGCCGCATAG |
| 295 | tetA(P) | Tetracycline | ARG | GGAAACCTTAGTTCAGTGACTTGG | CCCATTTAACCACGCACTGAA |
| 296 | tetB | Tetracycline | ARG | AGTGCGCTTTGGATGCTGTA | AGCCCCAGTAGCTCCTGTGA |
| 297 | tetB(P) | Tetracycline | ARG | TGGGCGACAGTAGGCTTAGAA | TGACCCTACTGAAACATTAGAAATATACCT |
| 298 | tetC | Tetracycline | ARG | ACTGGTAAGGTAAACGCCATTGTC | ATGCATAAACCAGCCATTGAGTAAG |
| 299 | tetD | Tetracycline | ARG | AATTGCACTGCCTGCATTGC | GACAGATTGCCAGCAGCAGA |
| 300 | tetE | Tetracycline | ARG | TTGGCGCTGTATGCAATGAT | CGACGACCTATGCGATCTGA |
| 301 | tetG | Tetracycline | ARG | TCGCGTTCCTGCTTGCC | CCGCGAGCGACAAACCA |
| 302 | tetH | Tetracycline | ARG | TTTGGGTCATCTTACCAGCATTAA | TTGCGCATTATCATCGACAGA |
| 303 | tetJ | Tetracycline | ARG | CAGCGCCCATACGCCATTTA | CCTACTTCAGTAGTGTGCCAAGC |
| 304 | tetK | Tetracycline | ARG | CAGCAGTCATTGGAAAATTATCTGATTATA | CCTTGTACTAACCTACCAAAAATCAAAATA |
| 305 | tetL | Tetracycline | ARG | ATGGTTGTAGTTGCGCGCTATAT | ATCGCTGGACCGACTCCTT |
| 306 | tetM | Tetracycline | ARG | GGAGCGATTACAGAATTAGGAAGC | TCCATATGTCCTGGCGTGTC |
| 307 | tetO | Tetracycline | ARG | TTGACGCTCCAAATTCATTGTATC | CAACATTAACGGAAAGTTTATTGTATACCA |
| 308 | tetPB | Tetracycline | ARG | TGGCAAGACGAGTTTGACTGA | GATCGCTCCACTTCAGCGATAA |
| 309 | tetQ | Tetracycline | ARG | TCGTTCATGCGGATATTATCAGAAT | CGCCTCAGAAGTAAGTTCATACACTAAG |
| 310 | tetR | Tetracycline | ARG | CCGTCAATGCGCTGATGAC | GCCAATCCATCGACAATCACC |
| 311 | tetS | Tetracycline | ARG | TTAAGGACAAACTTTCTGACGACAT | TGTCTCCCATTGTTCTGGTTCA |
| 312 | tetT | Tetracycline | ARG | CCATATAGAGGTTCCACCAAATCC | TGACCCTATTGGTAGTGGTTCTATTG |
| 313 | tetU | Tetracycline | ARG | GTGGCAAAGCAACGGATTG | TGCGGGCTTGCAAAACTATC |
| 314 | tetW | Tetracycline | ARG | ATGAACATTCCCACCGTTATCTTT | ATATCGGCGGAGAGCTTATCC |
| 315 | tetX | Tetracycline | ARG | AAATTTGTTACCGACACGGAAGTT | CATAGCTGAAAAAATCCAGGACAGTT |
| 316 | TLAbeta-lac | β-Lactamase | ARG | ACACTTTGCCATTGCTGTTTATGT | TGCAAATTTCGGCAATAATCTTT |
| 317 | Tn3 | MGE | MGE | GCTGAGGTGTTCAGCTACATCC | GCTGAGGTAGTCACAGGCATTC |
| 318 | TN5 | Insertional | MGE | CAGCATAAAAAATCCCGACAACA | CCCCGCAACAGACATACGT |
| 319 | TN5403 | MGE | MGE | AAGCGAATGGCGCGAAC | CGCGCAGGGTAAACTGC |
| 320 | tnpA-1 | Transposase | MGE | GCCGCACTGTCGATTTTTATC | GCGGGATCTGCCACTTCTT |
| 321 | tnpA-2 | Transposase | MGE | CCGATCACGGAAAGCTCAAG | GGCTCGCATGACTTCGAATC |
| 322 | tnpA-3 | Transposase | MGE | GGGCGGGTCGATTGAAA | GTGGGCGGGATCTGCTT |
| 323 | tnpA-4 | Transposase | MGE | CATCATCGGACGGACAGAATT | GTCGGAGATGTGGGTGTAGAAAGT |
| 324 | tnpA-5 | Transposase | MGE | GAAACCGATGCTACAATATCCAATT | CAGCACCGTTTGCAGTGTAAG |
| 325 | tnpA-6 | Transposase | MGE | TGCAGATGGTTTAACCTTGGATATTT | TCGGTTCATCAAACTGCTTCAC |
| 326 | tnpA-7 | Transposase | MGE | AATTGATGCGGACGGCTTAA | TCACCAAACTGTTTATGGAGTCGTT |
| 327 | tolC | Multidrug | ARG | GGCCGAGAACCTGATGCA | AGACTTACGCAATTCCGGGTTA |
| 328 | Tp614 | Transposase | MGE | GGAAATCAACGGCATCCAGTT | CATCCATGCGCTTTTGTCTCT |
| 329 | ra-A | Plasmid | MGE | AAGTGTTCAGGGTGCTTCTGCGC | GTCATGTACATGATGACCAAAA |
| 330 | raN | Plasmid | MGE | GCTTGGCGGTCAGCAATT | TTAGGAATAACAATCGCTACACCTTTA |
| 331 | trb-C | Plasmid | MGE | CGGYATWCCGSCSACRCTGCG | GCCACCTGYSBGCAGTCMCC |
| 332 | trfa | Transposase | MGE | ACGAAGAAATGGTTGTCCTGTTC | CGTCAGCTTGCGGTACTTCTC |
| 333 | ttgA | Multidrug | ARG | ACGCCAATGCCAAACGATT | GTCACGGCGCAGCTTGA |
| 334 | ttgB | Multidrug | ARG | TCGCCCTGGATGTACACCTT | ACCATTGCCGACATCAACAAC |
| 335 | vanA | Glycopeptide | ARG | GGGCTGTGAGGTCGGTTG | TTCAGTACAATGCGGCCGTTA |
| 336 | vanB | Glycopeptide | ARG | TTGTCGGCGAAGTGGATCA | AGCCTTTTTCCGGCTCGTT |
| 337 | vanC | Glycopeptide | ARG | CCTGCCACAATCGATCGTT | CGGCTTCATTCGGCTTGATA |
| 338 | vanC2_vanC3 | Glycopeptide | ARG | TGACTGTCGGTGCTTGTGA | GATAGAGCAGCTGAGCTTGTTC |
| 339 | vanD | Glycopeptide | ARG | GCCGGATTTTGTGATTCCAA | CAGAGGAACATAATGTTTCGATAAAATCT |
| 340 | vanG | Glycopeptide | ARG | TGTTTCGCAGAACCGTGTCAA | CCCTGCACTGTTCCATCTTCTC |
| 341 | vanHB | Glycopeptide | ARG | GAGGTTTCCGAGGCGACAA | CTCTCGGCGGCAGTCGTAT |
| 342 | vanHD | Glycopeptide | ARG | GTGGCCGATTATACCGTCATG | CGCAGGTCATTCAGGCAAT |
| 343 | vanRA | Glycopeptide | ARG | CCCTTACTCCCACCGAGTTTT | TTCGTCGCCCCATATCTCAT |
| 344 | vanRB | Glycopeptide | ARG | GCCCTGTCGGATGACGAA | TTACATAGTCGTCTGCCTCTGCAT |
| 345 | vanRC | Glycopeptide | ARG | TGCGGGAAAAACTGAACGA | CCCCCCATACGGTTTTGATTA |
| 346 | vanRC4 | Glycopeptide | ARG | AGTGCTTTGGCTTATCTCGAAAA | TCCGGCAGCATCACATCTAA |
| 347 | vanRD | Glycopeptide | ARG | TTATAATGGCAAGGATGCACTAAAG | CGTCTACATCCGGAAGCATGA |
| 348 | vanSA | Glycopeptide | ARG | CGCGTCATGCTTTCAAAATTC | TCCGCAGAAAGCTCAATTTGTT |
| 349 | vanSB | Glycopeptide | ARG | GAAGATAAAGAGGGAAGCGTACTC | CCGAATTGTCAGCCCTTGATAA |
| 350 | vanSC | Glycopeptide | ARG | ATCAACTGCGGGAGAAAAGTCT | TCCGCTGTTCCGCTTCTT |
| 351 | vanTC | Glycopeptide | ARG | ACAGTTGCCGCTGGTGAAG | CGTGGCTGGTCGATCAAAA |
| 352 | vanTE | Glycopeptide | ARG | GTGGTGCCAAGGAAGTTGCT | CGTAGCCACCGCAAAAAAAT |
| 353 | vanTG | Glycopeptide | ARG | CGTGTAGCCGTTCCGTTCTT | CGGCATTACAGGTATATCTGGAAA |
| 354 | vanWB | Glycopeptide | ARG | CGGACAAAGATACCCCCTATAAAG | AAATAGTAAATTGCTCATCTGGCACAT |
| 355 | vanXA | Glycopeptide | ARG | TCGTTGGGACGCTAAATATGC | GGACGGTAACCGTCCCATA |
| 356 | vanXB | Glycopeptide | ARG | AGGCACAAAATCGAAGATGCTT | GGGTATGGCTCATCAATCAACTT |
| 357 | vanYB | Glycopeptide | ARG | GGCTAAAGCGGAAGCAGAAA | GATATCCACAGCAAGACCAAGCT |
| 358 | vanYD | Glycopeptide | ARG | AAGGCGATACCCTGACTGTCA | ATTGCCGGACGGAAGCA |
| 359 | vatA | MLSB | ARG | ATGAACGGAGCGAATCATCGG | CCATACCGATCCAAACGTCATTTC |
| 360 | vatB | MLSB | ARG | GCAATTGTTGCTGCGAATTCAG | GTGCTGACCAATCCCACCA |
| 361 | vatE | MLSB | ARG | GACCGTCCTACCAGGCGTAA | TTGGATTGCCACCGACAATT |
| 362 | VEBbeta-lac | β-Lactamase | ARG | CCCGATGCAAAGCGTTATG | GAAAGATTCCCTTTATCTATCTCAGACAA |
| 363 | vgaA | Multidrug | ARG | GGAAGCTATAGAGGCGTTTGAATC | CCGAAGGTTCAATACTCAATCGAC |
| 364 | vgaALC | Multidrug | ARG | GTGAAGATGTCTCGGGTACAATTG | GAAATACCAGGATTCCCATGCAC |
| 365 | vgaB | Multidrug | ARG | TAAAAGAGAATAAGGCGCAAGGA | TGTTTAGTAGCATGTTGCATTTTCC |
| 366 | VIMbeta-lac | β-Lactamase | ARG | GCACTTCTCGCGGAGATTG | CGACGGTGATGCGTACGTT |
| 367 | 16S rRNA | Taxonomic |  | GGGTTGCGCTCGTTGC | ATGGYTGTCGTCAGCTCGTG |

**Table S4. Primers and amplification information for real-time qPCR**

| Gene name | Drug Class | Primers | | Standard curve formula | Amplification efficiency | *R^2^* | Amplification protocols |
| --- | --- | --- | --- | --- | --- | --- | --- |
| aadA17 | Aminoglycoside | F | TGTACGGCTCCGCAGTG | y = -3.3911x + 49.496 | 97.19% | 0.9975 | 1 min at 95ºC, following by 35 cycles of 10s at 95ºC, 20s at 58ºC, 20s at 72ºC |
|  |  | R | CACGGAATGATGTCGTCGTG |  |  |  |  |
| aadA21 | Aminoglycoside | F | ACGGCTCCGCAGTGGAT | y = -3.3777x + 50.256 | 97.72% | 0.9991 |  |
|  |  | R | GGCCACAGTAACCAACAAATCA |  |  |  |  |
| aph(3'')-Ia | Aminoglycoside | F | TAACAGCGATCGCGTATTTCG | y = -3.3362x + 53.333 | 99.41% | 0.9964 |  |
|  |  | R | TCCGACTCGTCCAACATCAATA |  |  |  |  |
| blaCARB | β-Lactamase | F | TGATTTGAGGGATACGACAACTCC | y = -3.2133x + 49.743 | 104.74% | 0.9996 |  |
|  |  | R | CTGTAATACTCCGAGCACCAA |  |  |  |  |
| qacH_351 | Multidrug | F | GTCGGTGTTGCTTATGCAGTCT | y = -3.3302x + 52.003 | 99.66% | 0.9992 |  |
|  |  | R | CAACCAGGCAATGGCTGTAA |  |  |  |  |
| msr(E) | Multidrug | F | CGGCAGATGGTCTGAGCTTAAA | y = -3.3216x + 48.795 | 100.01% | 0.9997 |  |
|  |  | R | CGCACTCTTCCTGCATAAAGGA |  |  |  |  |
| tetM | Tetracycline | F | GGAGCGATTACAGAATTAGGAAGC | y = -3.3132x + 52.269 | 100.37 | 0.999 |  |
|  |  | R | TCCATATGTCCTGGCGTGTC |  |  |  |  |
| floR | Others | F | AACCCGCCCTCTGGATCA | y = -3.2062x + 47.834 | 105.07% | 0.997 |  |
|  |  | R | GCCGTCGAGAAGAAGACGAA |  |  |  |  |
| ErmF | Others | F | CAGCTTTGGTTGAACATTTACGAA | y = -3.3747x + 49.797 | 97.84% | 0.9988 |  |
|  |  | R | AAATTCCTAAAATCACAACCGACAA |  |  |  |  |
| dfrA14 | Others | F | CGGATCATGTCATTGTTTCAGG | y = -3.1609x + 51.065 | 107.19% | 0.9949 |  |
|  |  | R | ATGTTAGAGGCGAAGTCTTGG |  |  |  |  |
| dfrA1 | Others | F | GGAATGGCCCTGATATTCCA | y = -3.1702x + 48.786 | 106.75% | 0.9975 |  |
|  |  | R | AGTCTTGCGTCCAACCAACAG |  |  |  |  |
| IS6100 | MGE | F | CGCACCGGCTTGATCAGTA | y = -3.3277x + 48.831 | 99.76% | 0.9996 |  |
|  |  | R | CTGCCACGCTCAATACCGA |  |  |  |  |
| intI1_337old | MGE | F | GCCTTGATGTTACCCGAGAG | y = -3.3154x + 48.306 | 100.27% | 0.9967 |  |
|  |  | R | GATCGGTCGAATGCGTGT |  |  |  |  |
| IS26 | MGE | F | ATGGATGAAACCTACGTGAAGGTC | y = -3.3876x + 50.029 | 97.33% | 0.9997 |  |
|  |  | R | CGGTACTTAATCTGTCGGTGTTCA |  |  |  |  |
| IS1247 | MGE | F | CGGCCGTCACTGACCAA | y = -3.2493x + 48.983 | 103.12% | 0.9999 |  |
|  |  | R | TCGGCAGGTTGGTGACG |  |  |  |  |
| ISSm2-Xanthob | MGE | F | TGGATCGACCGGTTCCAT | y = -3.2741x + 48.693 | 102.04% | 0.9956 |  |
|  |  | R | GCTGACCGAGCTGTCCATGT |  |  |  |  |
| QepA_1_2 | Others | F | GGGCATCGCGCTGTTC | y = -3.1996x + 45.079 | 105.37% | 0.9965 | 1 min at 95ºC, following by 35 cycles of 10s at 95ºC, 20s at 62ºC, 20s at 72ºC |
|  |  | R | GCGCATCGGTGAAGCC |  |  |  |  |
| sul1 | Others | F | CACCGGAAACATCGCTGCA | y = -3.3261x + 46.89 | 99.83% | 0.9999 | 1 min at 95ºC, following by 39 cycles of 5s at 95ºC, 30s at 60ºC |
|  |  | R | AAGTTCCGCCGCAAGGCT |  |  |  |  |
| bacteria |  | F | ACTCCTACGGGAGGCAGCAG | y = -3.2465x + 48.372 | 103.24% | 0.9999 |  |
|  |  | R | ATTACCGCGGCTGCTGG |  |  |  |  |

**Table S5. Carbohydrates used in the validation test**

| Resource diversity | D-Ribose  (CAS: 50-69-1 ) | D-galactose  (CAS: 59-23-4) | Glucose  (CAS: 50-99-7) | Sucrose  (CAS: 57-50-1) | Cellobiose  (CAS: 528-50-7) | D-Manntitol  (CAS: 69-65-8) | Myoinositol  (CAS: 87-89-8) | D-Fructose  (CAS: 57-48-7) | D-Xylose  (CAS: 58-86-6) | Maltose  (CAS: 6363-53-7) | D-Mannose  (CAS: 3458-28-4) | L-Arabinose  (CAS: 87-72-9) |
| --- | --- | --- | --- | --- | --- | --- | --- | --- | --- | --- | --- | --- |
| 1 |  |  |  |  |  |  |  |  | + |  |  |  |
| 1 | + |  |  |  |  |  |  |  |  |  |  |  |
| 1 |  |  |  |  |  |  | + |  |  |  |  |  |
| 1 |  | + |  |  |  |  |  |  |  |  |  |  |
| 1 |  |  | + |  |  |  |  |  |  |  |  |  |
| 3 |  |  | + | + |  |  |  |  |  |  |  | + |
| 3 |  |  |  |  |  | + | + | + |  |  |  |  |
| 3 | + |  |  |  |  |  |  |  | + | + |  |  |
| 3 | + |  |  |  | + |  |  |  |  |  | + |  |
| 3 |  | + |  |  |  | + |  |  | + |  |  |  |
| 6 |  |  |  | + |  | + | + |  |  | + | + | + |
| 6 |  | + |  | + |  | + |  | + | + |  |  | + |
| 6 | + |  | + |  | + |  |  |  | + | + | + |  |
| 6 |  | + | + |  | + | + |  | + |  | + |  |  |
| 6 | + | + | + |  |  |  | + | + | + |  |  |  |

Note: “+” represents the included compounds. All the compounds were randomly selected by the *sample* function in R software. All the chemicals were purchased from Sigma-Aldrich, Shanghai, China.
